# Deference or sociability? Insights from dogs’ and wolves’ human-directed behavior in a food-conflict task

**DOI:** 10.3389/fpsyg.2026.1854682

**Published:** 2026-06-30

**Authors:** Svenja Capitain, Sarah Marshall-Pescini, Gwendolyn Wirobski, Tabea Teichmann, Friederike Range

**Affiliations:** 1 Domestication Lab, Konrad Lorenz Institute of Ethology, University of Veterinary Medicine Vienna, Vienna, Austria; 2 Comparative Cognition Group, Institute of Biology, Faculty of Science, Université de Neuchâtel, Neuchâtel, Switzerland

**Keywords:** conflict behavior, dog, domestication, human–animal interaction, tractability, wolf

## Abstract

Whether dogs’ pronounced human proximity-seeking is motivated by a selection for hypersociability or deferential behavior (i.e., will to please the human) remains contested. Since disentangling these motivations has been challenging in amicable contexts, this study investigated the human-directed behavior of human-socialized dogs (*n* = 11) and wolves (*n* = 12) in a resource conflict, wherein a bonded human engaged in a tug of war over food with the animal while protesting loudly. Non-conflict trials served as controls. The deferential behavior hypothesis would be supported if dogs engaged less in conflict compared to non-conflict trials and to wolves, while conflict should not impact dogs if hypersociability drives them primarily. Results were mixed: dogs engaged less vigorously during the conflict than wolves but approached the task quicker. This suggests dogs may have been conflicted between the compliance of participating in the trained task and complying with the protesting human. Moreover, dogs showed more human directedness than wolves. While both species approached the human faster after conflict (suggesting reconciliation), the self-directed and adverse behaviors did not clearly indicate whether the animals perceived the situation as a strong conflict. To more definitively tease the two hypotheses apart, future studies should use stronger conflict scenarios and avoid pre-trained tasks.

## 1 Introduction

The close bond between humans and dogs has been a subject of fascination for centuries (Darwin, 1871; Miklósi, 2014; Sykes et al., 2020). One of the most notable traits our companion dogs are cherished for is their highly social, amicable behavior (Boada and Wirobski, 2024; Protopopova and Wynne, 2014; King et al., 2009; Diverio et al., 2016). Comparisons between dogs and wolves—the closest wild, present-day relatives to dogs’ ancestors—underline that dogs show more affiliative behavior towards humans, gazing at them more (Gácsi et al., 2005) and staying for longer in proximity (Bentosela et al., 2016; Capitain et al., 2025) and body contact than wolves do (Lazzaroni et al., 2020b).

Concordantly, research into the mechanisms of domestication has suggested that the process may have selected for a reduced fear of humans in dogs (Trut, 1999; Belyaev, 1979) (but see Range and Marshall-Pescini, 2022a) and an exaggerated motivation to seek social contact (hypersociability hypothesis: vonHoldt et al., 2017). This increased attraction has been shown towards humans familiar (Lazzaroni et al., 2020b; vonHoldt et al., 2017; Wirobski et al., 2021) and unfamiliar (Salomons et al., 2021; Bentosela et al., 2016) to the animals. Besides an increased attraction to humans, hypersociability may have conferred a selective advantage during domestication, as individuals that stayed near humans were less likely to backcross with wolves. Genetic studies revealed that dogs present a significantly higher occurrence of the GEN2P deletion compared to wolves, which was predictive of more socially affiliative behavior (vonHoldt et al., 2017; Tandon et al., 2024; vonHoldt et al., 2010) (though see Range and Marshall-Pescini, 2022b for critique on the studies’ design). Remarkably, this genetic variant underlies the William Beuren Syndrome in humans, a condition characterized—among other features—by exuberant gregariousness and lack of social inhibition (Järvinen et al., 2013).

However, looking at the present-day animals’ behavior during human interactions in more detail suggests that the situation is more complex. In addition to behaviors interpreted as affiliation, dogs usually also display more self-directed behaviors than wolves both towards bonded and familiar humans in greeting situations (Wirobski et al., 2021; Burkhard et al., 2023). These self-directed behaviors are commonly associated with stress (Hekman et al., 2012), appeasement (Kuhne et al., 2014; Pedretti et al., 2023a), or submission (Mech and Boitani, 2003). Moreover, dogs also showed significantly more ambiguous and submissive ear postures when greeting familiar humans compared to wolves (Capitain et al., 2025). These observations suggest that dogs might perceive these interactions at least partly as a dominance interaction where they affirm their submissive status towards humans and tolerate physical contact even when they feel conflicted or stressed. Considering that humans have full control over the dogs’ resources (in the tested populations) and that human attention arguably presents one—if not the—most important resource for pet dogs (Bakos et al., 2025), Wynne (2021) suggested that humans occupy a role of “super-dominance” in dog-human relationships. Therein, dogs’ heightened sensitivity to social hierarchy may contribute to their outstanding willingness to accept human leadership, further explain the intriguing human-proximity-seeking pattern we see compared to wolves who only tend to approach humans when they feel in control of the situation (Wynne, 2021).

These observations align with an alternative explanation: The deferential behavior hypothesis postulates that dogs may have been selected for compliance and tractability, with a stronger inclination to please and accept the human as the leader (Range et al., 2019). Evidence for this comes from differences in dogs’ and wolves’ willingness to be physically restrained by a human (Zimen, 1987; Wirobski et al., 2025), to wait for the human to take the lead (Range et al., 2019), to follow humans’ commands (Vasconcellos et al., 2016) and to let go of a resource when the human asked them to (Ujfalussy et al., 2020). From an evolutionary perspective, early dogs that were more amendable to human control would have been easier to manage and less dangerous, while the ones challenging humans may have been discarded promptly.

In addition to a specific selection by humans, the different perception of humans may have also been a consequence of the change in the socio-ecology during the domestication process that led to different dominance interactions between conspecifics. Unlike wolves, which evolved as cooperative hunters reliant on group coordination, dogs spent much of their evolutionary history as facultatively social scavengers (Majumder et al., 2014; Marshall-Pescini et al., 2017; Font, 1987; Berghänel et al., 2025). They need to compete for monopolizable, irregular resources and are less willing to accept subordinate conspecifics at a food source, which differs from more tolerant food-sharing in wolves (Range et al., 2015; Dale et al., 2017; Lazzaroni et al., 2017; Berghänel et al., 2025). This steeper social hierarchy in dogs is paralleled by differences in the species’ conflict strategies with conspecifics, with more frequent post-conflict reconciliation in the cooperation-dependent wolves, compared to more stringent distance-maintenance, or conflict-avoidance, strategies in the facultatively-social dogs (Cafazzo et al., 2018) (though there are signs of dogs showing reconciliation towards humans (Cavalli et al., 2016)). Intriguingly, one of the most overt signals to upkeep dominance hierarchies and thus reduce conflict among dogs and wolves is greeting behavior (Schilder et al., 2014), shown mainly from subordinates towards dominant conspecifics through actively submissive greeting (Cafazzo et al., 2010; Cafazzo et al., 2016)—a very similar behavior dogs show towards humans, as we discussed above.

Taken together, two opposing (though not mutually exclusive) drivers for the differences in dogs’ and wolves’ human-directed behavior have been proposed: selection for hypersociability (vonHoldt et al., 2017) and human and socioecological selection for deferential behavior (Range et al., 2019) (see also Marshall-Pescini et al., 2017; Wynne, 2021). However, these motivations have primarily been tested in amicable situations with human partners (Burkhard et al., 2023; Wirobski et al., 2021; Lazzaroni et al., 2020b; Bentosela et al., 2016), rendering them difficult to tease apart. During positive interactions, both a desire for social closeness and a wish to reaffirm social hierarchy can lead to the same proximity-seeking behaviors. Moreover, a recent study that aimed at understanding the underlying motivations behind dogs’ proximity-seeking behavior by analyzing the animals’ facial expressions was limited by the behavioral biases the humans showed towards the two species (Capitain et al., 2025). These limitations highlight the need for a more robust approach.

To address these gaps, we compared the human-directed behavior of similarly hand-raised and kept dogs and wolves in a more confrontational context, where the motivations can be more clearly distinguished. The human–animal conflict centered on a brief tug of war over food, which incentivized the animals’ approach and mirrored the general asymmetric resource dependency in human-(captive) canid relationships (Wynne, 2021). To represent the conflict, the human actively opposed the animal’s attempt to retrieve the food by protesting loudly. We compared the species’ engagement during the conflict compared to the non-conflict trials, as well as their human-directed behavior after the conflict, to understand how the two species react to human opposition in goal-directed context. In addition, we also coded the humans’ behavior to assure that biases in their actions towards either species were not the reason for the differences we expected to see between the species. If, following the hypersociability hypothesis, dogs approach purely out of a desire for social contact, a mild conflict with the human should leave the dogs rather unaffected. In contrast, if dogs’ proximity-seeking behaviors are at least partially motivated by a desire to avoid conflict and please a higher-ranking individual, as proposed by the deferential behavior hypothesis, dogs should become more avoidant than wolves’ when the interaction with the human turns into a conflict.

**Figure 1:**
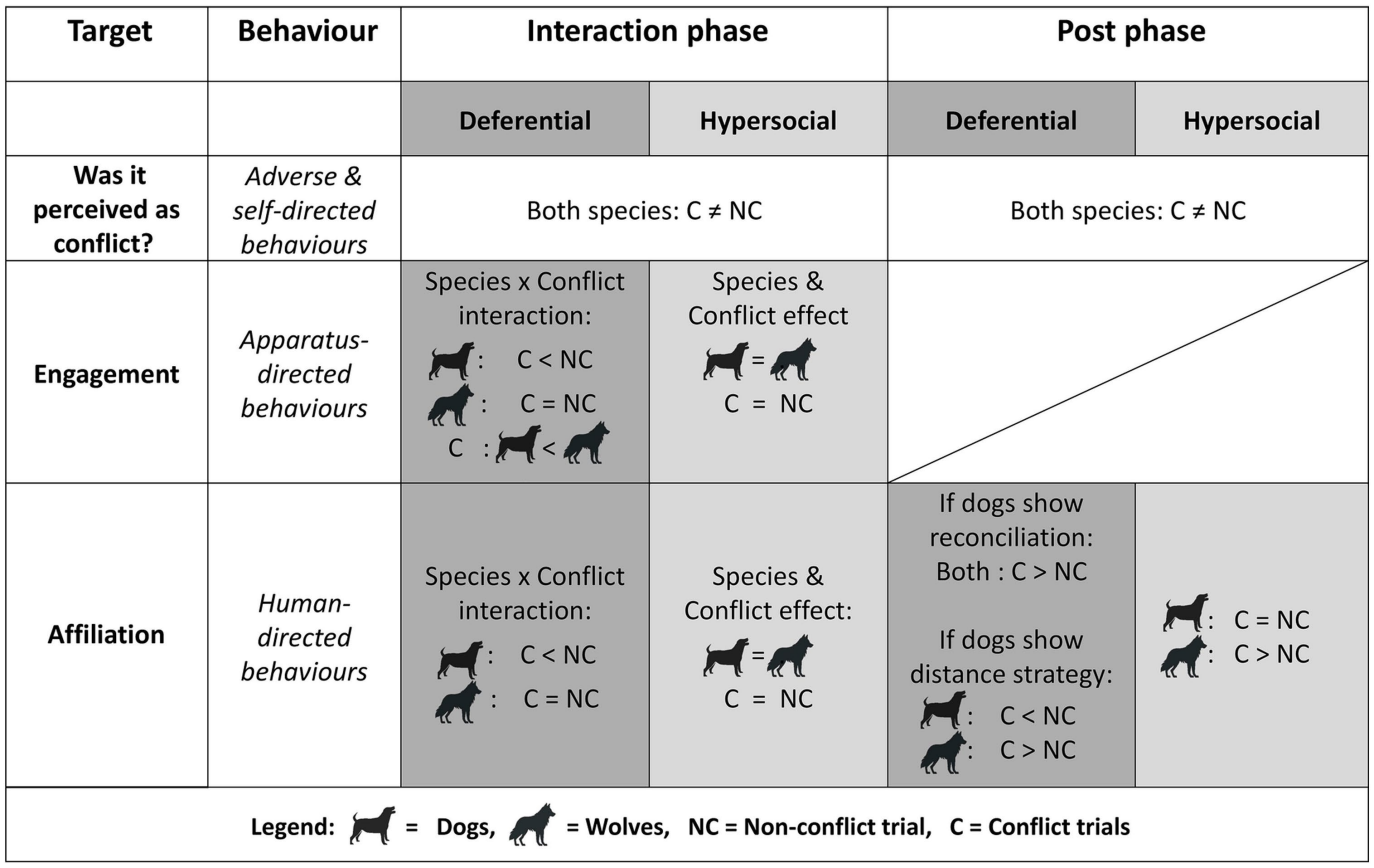
Hypotheses and prediction summary. The behavioral differences we would expect to see between species and between conflict and non-conflict trials in support of the hypersociability or the deferential behavior hypothesis.

Taking our study design into account, we summarized hypotheses and predictions in more detail in Figure 1. Based on the deferential behavior hypothesis, we expected an interaction effect between species and whether a conflict occurred or not. We predicted dogs to show reduced engagement during conflict trials compared to non-conflict trials and compared to wolves, reflecting a conflict-avoidance and compliance strategy. Additionally, if dogs employ distance maintenance strategies with humans as they were shown to do with conspecifics (Cafazzo et al., 2018), we expected more affiliation towards the human in the non-conflict trials compared to wolves, while this should drop after a conflict. Alternatively, we might see an increase in human-affiliation after conflict in both species mirroring reconciliation, especially if they “lost” the tug of war, as had been the case for wolves with conspecifics and dogs in a recent study with humans (Cavalli et al., 2016; Cafazzo et al., 2018). In support of the hypersociability hypothesis, we expect species differences without an interaction with the conflict condition given that the conflict is mild and affiliation would override subtle aversion (Wirobski et al., 2021; Capitain et al., 2025). Thus, we predicted higher affiliation towards the human in dogs compared to wolves regardless of conflict condition.

## 2 Materials and methods

### 2.1 Ethical statement

The study received ethical approval from the ‘Ethik und Tierschutzkommission’ of the University of Veterinary Medicine Vienna (Reference Number: ETK-117/07/2023). All methods were carried out in accordance with Good Scientific Practice guidelines and national legislation. All methods are reported in accordance with ARRIVE guidelines. The procedures adhere to the tenets of the Declaration of Helsinki. This experiment does not fall under the requirement for human ethics (or human accordance) approval. The trainer participants gave informed consent to take part in the study and also provided informed consent for the use of the videos included in the Supplementary material.

### 2.2 Study subjects

Thirteen wolves and 11 dogs housed at the Core Facility Wolf Science Center (CF-WSC) in Ernstbrunn, Austria, started taking part in the study. One wolf was excluded in the training due to fear of the apparatus, leaving 12 wolves (7 males, 5 females, age 1–14 years) and 11 dogs (5 males, 6 females, age 1–9 years, all mongrels) for the test sessions and analysis. At the CF-WSC, all canids are hand-raised from an early age and live in small conspecific groups with daily human contact. This ensures that wolves and dogs have similar experiences with humans throughout their lives, allowing for a fair comparison and, in this case, the possibility to create a safe conflict under comparable, controlled conditions. The two female trainers involved as conflict partners in the study have hand-raised the animals or worked with them for at least half the animal’s life (at least 5 years). Hence they were deeply familiar and strongly bonded with the individuals of both species (Burkhard et al., 2023), increasing the likelihood of the conflict being seen as severe enough to necessitate reconciliation behavior (Cools et al., 2008; Cafazzo et al., 2018).

### 2.3 Apparatus and study setup

**Figure 2:**
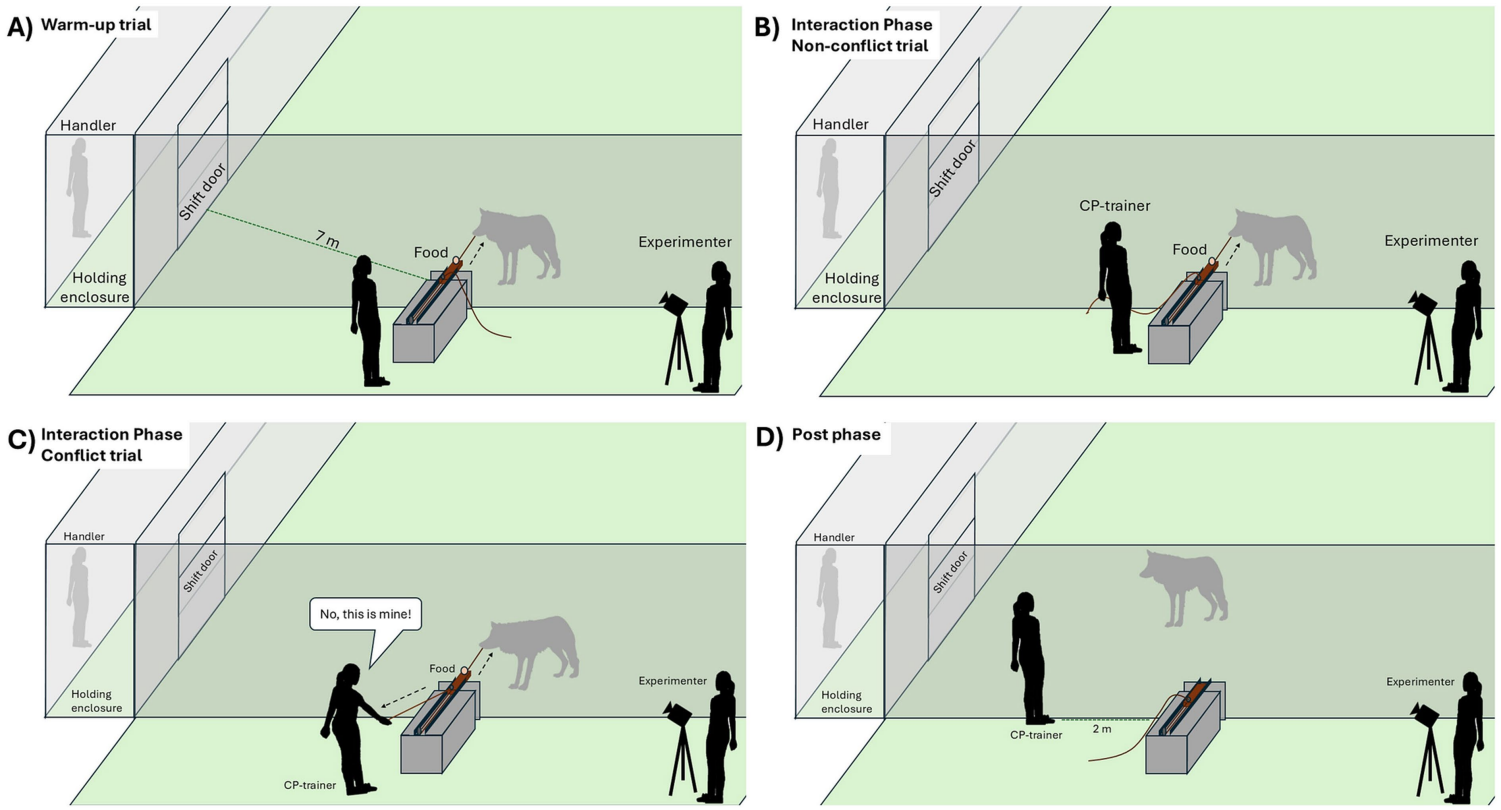
Study set up across trials. The position of the trainer acting as conflict partner (CP-trainer), experimenter, handler, apparatus and animal during **(A)** the first two warm-up trials, **(B)** the Interaction phase of the non-conflict trial, the **(C)** the Interaction phase of the two conflict trials, and **(D)** the Post phase that follows the Interaction phase in the non-conflict and conflict trials. The black dashed line indicates the pulling direction(s) at the rope to get the food.

To mimic a food-conflict situation, the apparatus consisted of a wooden box (l × h × w: 60 cm × 30 cm × 40 cm) on top of which a wooden board, attached to a rope on either end, could be moved back and forth inside a guiding rail (Figure 2). While the apparatus was located outside the enclosure, one rope extended into the enclosure to allow the animal to pull the wooden board with a piece of food on top towards them. The rope at the other end of the movable board ran through a 3:1 pully system away from the fence, allowing the trainer leverage to pull against the animal. By pulling on the rope, either party could hence pull the food to their side and eat without the conflict partner getting any access. If both pulled at the same time, a tug of war over the food ensued.

Each animal was trained and tested individually in the familiar test enclosure with water at libitum. The apparatus was located right outside the fence, seven meters from the shifting door that allowed the animal to pass from the holding enclosure into the shifting enclosure in each trial. The shifting door was manually operated by a conflict-uninvolved trainer outside the enclosure. For easier differentiation between the trainer at the shift and the trainer involved in the conflict, we will refer to the former as “handler,” and the latter as conflict partner trainer “CP-trainer” in the following paragraphs. The experimenter was always located four meters to the side and behind the apparatus to film the test, instruct the trainers, and replenish the food on the apparatus (Figure 2). The CP-trainer stood slightly behind or next to the apparatus, depending on the trial (see Procedure).

### 2.4 Study procedure and test conditions

The study consisted of training and test sessions.

#### 2.4.1 Training

Each animal was trained with positive reinforcement (food reward and clicker) by a conflict-uninvolved trainer to operate the apparatus by pulling the rope to get the food. Training started close up to the apparatus, increasing the distance in three steps (0.5 m distance, 2 m distance, 7 m distance (from inside the holding enclosure)) whenever the animal successfully pulled the food in three times in a row. The final training threshold consisted of seven consecutive trials with the CP-trainer standing next to the apparatus and the animal starting from the holding enclosure. The goal was to familiarize the animal with the paradigm that the human can get the food as well so they would not be confused during the test trials. In four trials, the animal pulled out the food, and in three interspacing trials, the human pulled out the food before the animal could get to the apparatus, with the human turning away as if they had not seen the animal. The animals took on average 2.6 sessions (min. 1, max. 5) to do so.

#### 2.4.2 Test sessions

Each animal completed five test sessions, with at least 5 days between each session to reduce carry-over and habituation effects. For each individual, the CP-trainer engaged in the conflict was the same across all trials.

At the beginning of each test session, the animal was allowed to freely explore the enclosure until they stopped sniffing around and came to a halt near the shifting door. The handler called the animal into the holding enclosure and closed the shifting door. Each test session consisted of five consecutive trials, counterbalanced by whether the animal or human received the food (for an overview, see Table 1 and S1 Video).

**Table 1:** Test session overview.

Variable	Trial (frequency)		
	Warm-up (*n* = 2)	Non-conflict (*n* = 1)	Conflict (*n* = 2)
Food	Always the animal	Animal or human (counterbalanced)	1× animal, 1× human (order counterbalanced)
Conflict during interaction-phase?	No	No	Yes
Post phase (2 min.)	No	Yes	Yes

Each animal participated in five test sessions, each consisting of five consecutive trials (left to right). The table indicates for each of the three trial types who “won” the food, whether there was a conflict (3 s of tug of war, human protests during and right after the conflict) over the food, and whether the human stayed at the fence for 2 min after the food was eaten (Post phase) in this trial. Who received the food was counterbalanced between human and animal dyads across the trials and five test sessions. The physical set up for each trial is displayed in Figure 2.

##### 2.4.2.1 Two warm-up trials

The CP-trainer quietly stood behind the apparatus without touching it. On the experimenter’s verbal signal (“okay”), the handler opened the shift door and the animal was free to approach the apparatus, pull the rope and obtain the food (Figure 2A). Once, successful, the animal was called back by the handler, the experimenter replenished the food, and the same procedure was repeated once more. These trials had two goals: (1) Verify that the animal was motivated to take part that day. If they did not pull the rope within 30s of the shift door opening, two times in a row, the session was restarted on a different day. (2) Build the expectation that the food would be easily attainable.

##### 2.4.2.2 One non-conflict trial

The CP-trainer stood next to the apparatus, with the rope in hand. On the experimenter’s verbal signal (“okay”), the handler opened the shift door, and the animal was free to approach the apparatus. The CP-trainer looked at the animal with a friendly face and remained quiet. It was predetermined who received the food. If it was the animal’s turn, the CP-trainer allowed the animal to simply pull the rope, without any resistance (Figure 2B, “Interaction phase”). Once the animal had eaten the food, the CP-trainer pulled the empty board back (to prevent the animal from damaging it or getting distracted by it), walked to a spot two meters to the left of the apparatus, and stood there neutrally, looking into the enclosure (“Post phase,” Figure 2D). They smiled at the animal if the animal approached, but did not interact with them further. Reason being that conspecific reconciliation interactions are generally one-sided (Lazzaroni et al., 2017), but this is seldomly the case in human–animal interactions. However, too much interaction might trigger a greeting-like situation. Hence, we chose this low-level acknowledgement. After 2 min, the experimenter gave the cue to the handler to call the animal back into the holding enclosure.

If the CP-trainer was predetermined to receive the food in the Interaction phase, they pulled the food towards them as soon as the animal passed the shifting door and pretended to eat the food, turning away, pretending they did not see the animal coming. Also in this case, the CP-trainer afterwards stood at the fence for 2 min for the Post phase.

##### 2.4.2.3 Two-conflict trials

The conflict trials commenced similarly to the non-conflict trials with the difference that a conflict ensued during the Interaction phase. Once the animal passed the shifting door, the CP-trainer started to lean forward with an angered expression, protesting loudly, telling the animal to go away and leave the apparatus alone. If the animal started pulling the rope, the CP-trainer pulled against them for 3 s (Figure 2C). Since the animals were not used to tug of war with the trainers and this does not seem to be a natural play behavior with humans (Capitain et al., 2024), we exclude the possibility that this was understood as play. If the animal was predetermined to get the food, the CP-trainer allowed the animal to pull the food into the enclosure, though they continued to argue loudly all the way until the animal had eaten the food and the CP-trainer moved the board back. Again, the Post phase commenced, where the CP-trainer went to stand at the fence for 2 min with a neutral expression. If the CP-trainer was predetermined to get the food, they pulled the board towards them and out of reach of the animal after the 3 s of tug of war were up. Pretending to eat the food and enjoy it, they turned away, slipping the food into their pocket, and moved to stand at the fence for 2 min. To note here is that the trainers wore their food west during the testing which always contained food, so the addition of this “pretend” food to the pocket could not be noted by smell by the animals. If the animal was supposed to get the food but stopped engaging before the tug of war was over, the CP-trainer continued arguing and staring at the animal. First statically for 5 s, then while slowly pulling the rope back for another 5 s. If the animal reengaged, it was free to pull the rope to its side. If it did not reengage, the CP-trainer followed the same fake-eating protocol as above, before moving to the fence for 2 min. At the end of the two-minute Post phase, the animal was called back and another conflict trial commenced. If the animal received the food in the first conflict trial, the CP-trainer received it in the second, and vice versa.

Since the work with the animals at the CF-WSC is built on positive interactions and trusting bonds, we did not want to jeopardize these bonds in this study. Consequently, we asked the trainers to use a more confrontational, louder voice than they usually ever would and a more threatening (i.e., forward leaning) body posture, but not to scream at the animals or use fast, punching movements towards them.

### 2.5 Behaviors coded

The whole test session was videotaped and the conflict and non-conflict trials were coded using the open source software BORIS (Behavioral Observation Research Interactive Software) (v.8.25.4, Friard and Gamba, 2016). During the Interaction phase, we measured the animal’s engagement with the apparatus, their human-directed behaviors, self-directed and adverse signals (Table 2). During the Post phase, human-directed behaviors as well as the self-directed and adverse signals were coded (Table 2).

**Table 2:** Animal ethogram.

Category	Behavior	Definition
Interaction phase interaction with the apparatus, from the moment the animal leaves the shift to the moment one of the parties has eaten the food		
Engagement with the apparatus	Proximity to apparatus (D)	At least one paw within 1 body length of the apparatus
	Pulling (D)	Rope in mouth, leaning backwards with tension on the rope
	Manipulating (D)	Touching or contact-sniffing the rope, excluding pulling
	Tucking at the rope (F)	Backwards movements while pulling the rope
	Pull initiations (F)	Number of times the animal starts to pull the rope
	Latency to get in proximity	Time from the moment two paws are out of the shift door to getting within 1 body length of the apparatus
	Latency to start pulling	Time from the moment two paws are out of the shift door to starting to pull
	Engagement (scaled)	1 = engage continuously, 2 = stops engaging (manipulation or pulling) before the 3 s are over, 3 = does not start engaging (never comes in proximity)
Self-directed behaviors	Nose lick (F)	Touches nose with the tongue (excluding 5 s after eating)
	Yawn (F)	Wide mouth stretch with long inhalation
	Shaking (F)	Rapidly moving the body from side to side
	Auto-grooming (F)	Licking/ scratching one’s skin or fur (excluding nose licks)
Adverse behaviors	Head dip (F)	Head and neck are lowered; ears are rearward while the subject’s gaze stays on the human
	Gaze aversion (F)	In response to having eye-contact with the human subject turns its head to the side orienting the gaze away
	Pacing (D/F)	Repetitive walking or trotting at a steady speed up and down, without any exploratory purpose or obvious focus on the surroundings, while potentially breathing with open mouth and lolling tongue, corners of the mouth are raised
	Growling (D/F)	Low-pitched rumbling
	Crouching (D/F)	Leaning backwards, bending the legs, lowering the tail (slightly wagging) or tucking the tail, rearward ears
	Leaving (F)	Walking out of proximity of the apparatus
	Whining (D/F)	High-pitched vocalization
Human-directed	Gazing at the human (D)	Head directed towards the human
	Tail wagging (D)	Tail is moved from side to side
Post phase after the interaction while the human stands friendly at the fence; after the human turns away from the apparatus to walk to the fence until 2 min later		
Human-directed	Gazing at the human (D)	Head directed towards the human
	Proximity to the human (D)	At least two paws within one body length of the fence part where the human is standing
	Tail wagging (D)	Tail is moved from side to side
	Latency to get in proximity of the human	From the moment the human turns away from the apparatus to walk away to the moment the animal gets within one body length of the human
Self-directed behaviors	See Interaction phase	
Adverse behaviors	See Interaction phase	

The animal behaviors that were coded during the Interaction and during the Post phase afterwards, measured as duration (D) or frequency (F).

To investigate whether the human’s behavior differed between species, the CP-trainers’ behavior during the Interaction phase was also analyzed to exclude possible influences. For that purpose, we measured the duration of gazing at the animal, leaning forward, pulling actively, talking to the animal, and the use of one or two hands while pulling. How aggressive the voice sounded was measured as relative duration on a three-point scale (1 = conversational, 2 = louder and rougher than their talking voice, possibly whiny (high pitched, nasal), 3 = harsh/growling tone, much louder than talking voice). All videos were coded with the animal covered by a black box, leaving the coder blind to the species.

Twenty percent of the videos were recoded by two additional researchers, one for the human and one for the animal videos respectively, resulting in a good interrater reliability (Rousson, 2011) for the animal videos (average Intraclass correlation coefficient (ICC) = 0.92) and the human videos (average ICC = 0.90) (for details see Supplementary material).

### 2.6 Statistical analysis

All statistical analyses were conducted in R (RStudio version 2023.06.0; Team R. C, 2023). To account for the different times spent in proximity or engaging with the apparatus, the apparatus-directed behaviors, tail wagging, and gazing at the human were analyzed as proportion of time spent in proximity of the apparatus, and the other non-apparatus-directed behaviors were analyzed as time spent in proximity but not pulling or manipulating the apparatus (since these behaviors excluded all other non-apparatus directed behaviors).

To test the effect of species and conflict on the displayed behaviors in the Interaction and Post phase respectively, generalized linear mixed models (GLMMs) (Baayen et al., 2008) using the packages lme4 (Bates et al., 2015) and glmmTMB (Brooks et al., 2017) were fitted. The interaction term as well as the main effects of Species (CF-WSC wolf, CF-WSC dog) and Conflict condition (Non-conflict, Conflict) were included as test predictors. The apparatus-directed behaviors were also analyzed with the factor Conflict trial (Non-conflict trial, Conflict trial 1, Conflict trial 2) rather than Conflict condition to extract whether experiencing a conflict in the previous trial had an effect. Likewise, models that explored apparatus-directed behaviors were ran on a dataset excluding the non-conflict trials where the human received the food, considering that apparatus-directed behaviors were not possible in these trials. In all models, animal age, animal sex, and session number were included as control predictors (Rosado et al., 2012; Head et al., 1997). Continuous numerical parameters were z-transformed to aid model stability, interpretability and convergence (Bates et al., 2015). Whether the human or the animal received the food was included as control predictor unless the behavior of interest purely took place before either party received the food (e.g., duration of pulling). Animal ID was added as random effect and random slopes were included where appropriate, with the respective factors manually dummy-coded and centered (see Statistical results, Supplementary material). Relative durations were analyzed with a beta-distribution and frequencies with a Poisson distribution with proximity or proximity minus pulling time as a log-transformed offset term. Cox proportional hazards models were used to test the latencies in the same model structure using the package coxme (Therneau, 2024).

Models were examined for overdispersion, distribution of residuals, best linear unbiased prediction (BLUPs), and multicollinearity where congruent with the model assumptions. Alternative distributions (negative binomial or binary) were employed wherever necessary (see Statistical results, Supplementary material). Furthermore, model stability was assessed by comparing the estimates obtained from the full model with those obtained from models with the levels of the random effects excluded one at a time. To keep the type I error rate at 5%, full-null model comparisons were conducted using a likelihood ratio test (Dobson and Barnett, 2018) with the null model lacking the test predictors, but comprising the control predictors and complete random effects structure. The model summaries of all factors are listed in the Supplementary material. Finally, Tukey-adjusted pairwise comparisons were employed using the emmeans package (Lenth and Piaskowski, 2026). Parametric bootstrapping was performed to obtain confidence intervals (function ‘boot.glmmtmb’ of glmmTMB), which are reported in the Supplementary material.

To test whether any of the human behaviors differed between species in the interaction phase, the same test statistics and model checks as above were used, with the main factor of interest being Species, session number (z-transformed) as control factor, and human and animal ID as random factors.

## 3 Results

### 3.1 Check 1: human influence

There was no influence of Species on the proportion (of trial duration) of gazing at the animal (*χ*^2^ = 2.29, df = 1, *p* = 0.13), talking (*χ*^2^ = 0, df = 1, *p* = 1.0), leaning forward (*χ*^2^ = 0.24, df = 1, *p* = 0.63), or holding the rope with one or two hands (*χ*^2^ = 0.002, df = 1, *p* = 0.97). Likewise, the human conflict partners did not differ by species in how aggressive their voice sounded (*χ*^2^ = 0.002, df = 1, *p* = 0.97). The human conflict partners spent more time pulling actively on the rope when in conflict with a wolf rather than a dog (*χ*^2^ = 6.78, df = 1, *p* = 0.009, Post-hoc: dogs vs. wolves: est. = −0.64, SE = 0.23, z-ratio = −2.76, *p* = 0.006; for confidence intervals see Supplementary material), though this was likely caused by the animals’ behavior (see Species differences).

### 3.2 Check 2: perception of the interaction as conflict

Since all of the animals’ self-directed and adverse reactions but nose licks were too rare to be analyzed by themselves (i.e., occurred in less than 10% of trials), we combined them into one summed variable, respectively. Hence the variable “self-directed behaviors” contains the summed occurrence of nose licks, yawns, and auto-grooming, while “adverse behaviors” contains crouching, leaving, gaze aversions, head dipping, growling, whining, and pacing (from most to least frequent occurrence).

**Figure 3:**
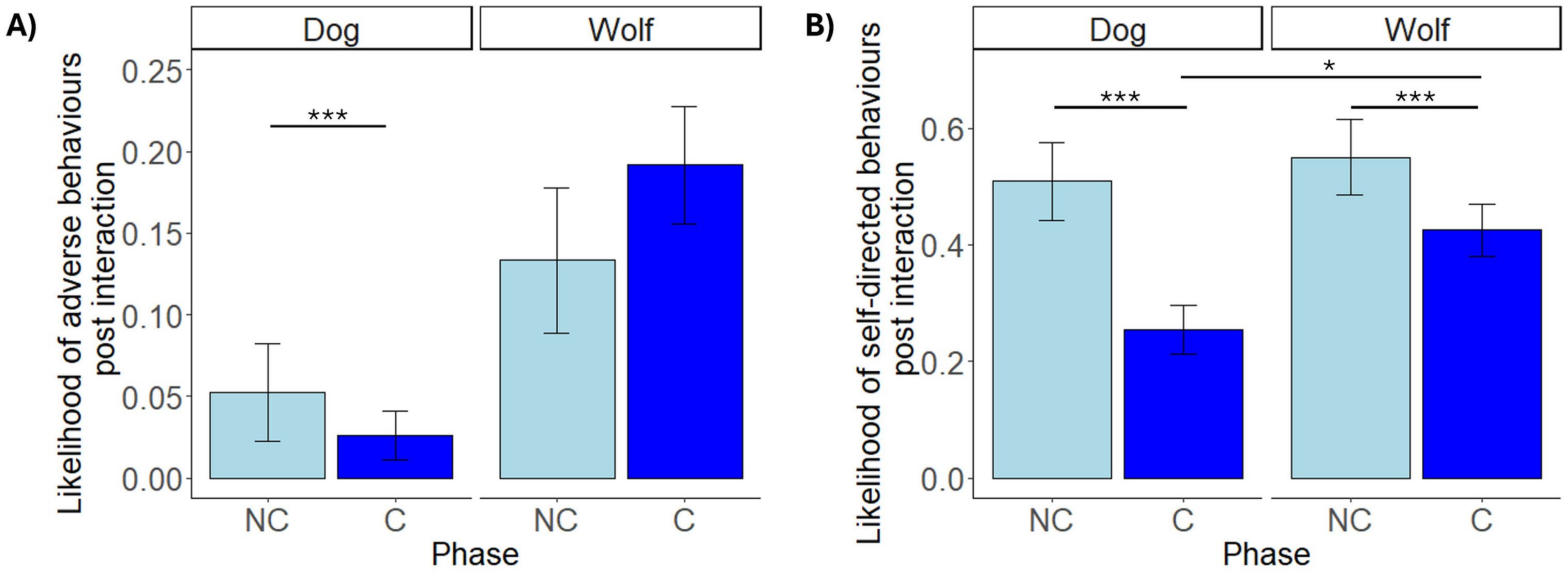
The likelihood of self-directed behaviors shown by dogs and wolves **(A)** during and **(B)** after a non-conflict (NC, light blue) or conflict (C, dark blue) interaction. The tails represent the standard error. Significant differences are indicated with asterisks above (^*^0.05 > *p* > 0.01, ^**^0.01 > *p* > 0.001, ^***^p < 0.001).

During the Interaction phase, there was no interaction effect of species and conflict and no effect of conflict condition itself on the likelihood of self-directed (Conflict effect: conflict vs. non-conflict condition: est. = −0.77, SE = 0.58, z-ratio = −2.47 *p* = 0.22; for confidence intervals see Supplementary material) (Figure 3A) or adverse behaviors (Full-null model: *χ*^2^ = 2.58, df = 3, *p* = 0.46). Likewise, after the conflict no interaction effects on self-directed (Species x Conflict effect: *χ*^2^ = 2.04, df = 3, *p* = 0.15) or adverse behavior (Full-null model: *χ*^2^ = 4.40, df = 3, *p* = 0.22) occurred. However, both species showed fewer self-directed behaviors after a conflict occurred compared to trials where no conflict took place (Conflict effect: est. = −1.35, SE = 0.31, z-ratio = −4.42 *p <* 0.01) (Figure 3B). While this seems to be the case during the interaction as well when looking at the graph (Figure 3A), there was no significant conflict effect on self-directed behaviors during the interaction (conflict vs. non-conflict condition: est. = −0.78, SE = 0.49, z-ratio = −1.58 *p* = 0.11). These results suggest that the animals perceived the two situations differently.

### 3.3 Species differences in relation to the conflict situation

To explore our hypotheses, we investigated the interaction effect of species and whether there was a conflict or not (Conflict condition), as well as whether there was an effect of previous conflict (Conflict trial) on the animals’ engagement and human-directed behavior. All variables apart from the latency were analyzed as proportion of being in proximity (1 body length) of the apparatus.

**Figure 4:**
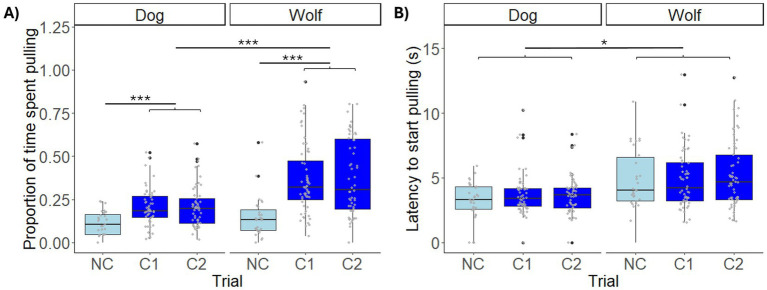
Effect of species and conflict on engagement during the interaction phase. **(A)** The proportion of time spent pulling the rope and **(B)** the latency to start pulling the rope in dogs and wolves in non-conflict trials (NC, light blue), the first conflict trial (C1, dark blue), and the second conflict trial (C2, dark blue). Each box represents the interquartile range (25th–75th percentile) of the behavior, with the median marked by the thick line. Whiskers extend to the smallest and largest values within 1.5 times the interquartile range. Individuals are represented as grey dots, or as black dots in case of outliers. Asterisks above illustrate the effect significance (^*^0.01 < *p* < 0.05, ^***^p < 0.001).

Starting with the apparatus-directed behaviors, the interaction of Species and Conflict condition revealed that while dogs and wolves did not differ in the proportion of time they spent pulling in the non-conflict trials, dogs pulled significantly less than wolves during the conflict trials (Species x Conflict condition: *χ*^2^ = 4.24, df = 1, *p* = 0.04, for Post-hoc details and confidence intervals see Supplementary material) (Figure 4A). In contrast, dogs manipulated (touched/sniffed without pulling) the rope more than wolves did, independent of conflict (Species effect: dogs vs. wolves: est. = 0.81, SE = 0.17, z-ratio = 4.8, *p* < 0.001). Moreover, dogs more frequently stopped and restarted pulling the rope than wolves (Species effect: dogs vs. wolves: est. = 1.16, SE = 0.18, z-ratio = 6.48, *p <* 0.001) and both did so more often in the non-conflict compared to the conflict condition (Conflict condition effect: conflict vs. non-conflict: est. = −1.13, SE = 0.14, z-ratio = −8.12, *p <* 0.001). There was no influence of whether it was the first or second conflict trial on how often the animal tugged at the rope during the conflict (Conflict trial effect: conflict trial 1 vs. 2: est. = 0.11, SE = 0.15, z-ratio = 0.73, *p* = 0.46), but wolves tugged more frequently than dogs during conflict trials overall (Species effect: dogs vs. wolves: est. = −0.96, SE = 0.32, z-ratio = −3.03, *p* = 0.002). The likelihood to completely disengage from the task was not affected by species or conflict (Species x Conflict condition: *χ*^2^ = 0.00, df = 1, *p* = 0.99). Only in three trials did an animal (all dogs) refuse to approach the apparatus again in the second conflict trial and the session had to be terminated. Finally, a Cox proportional hazards models revealed that dogs were faster than wolves to approach the apparatus (Species-effect (Dogs vs. wolves): *β* ± SE = −2.04 ± 0.41; *p* < 0.001) and pull the rope (Species-effect (Dogs vs. wolves): *β* ± SE = −1.24 ± 0.54; *p* = 0.03), regardless of previous conflict (Figure 4B).

**Figure 5:**
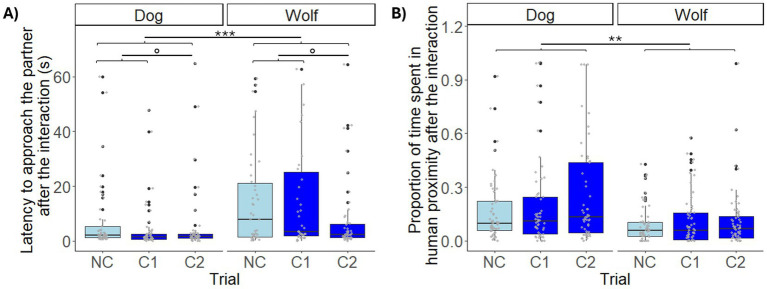
Effect of species and conflict on the human-directed variables after the interaction. **(A)** The latency to approach the human after one of the parties received the food and **(B)** the proportion of time the animals spent within one body length of the human after the interaction. Each box represents the interquartile range (25th–75th percentile) of the behavior, with the median marked by the thick line. Whiskers extend to the smallest and largest values within 1.5 times the interquartile range. Individuals are represented as grey dots, or as black dots in case of outliers. Asterisks above illustrate the effect significance (°*p* = 0.08, ^**^0.001 < *p* < 0.01, ^***^p < 0.001).

There were no interaction effects between species and conflict trial in the affiliative behaviors. While dogs wagged their tail more than wolves during the Interaction phase (Species effect: Dogs vs. wolves: est. = 2.11, SE = 0.52, z-ratio = 4.01, *p* < 0.001; for confidence intervals see Supplementary material), both species wagged more during the conflict compared to the non-conflict trials (Conflict condition effect: Non-conflict vs. conflict trials: est. = 0.27, SE = 0.11, z-ratio = 2.42, *p* = 0.02). Several variables influenced how fast the animals approached the human in the Post phase: both species were marginally faster in the second conflict trial compared to the non-conflict and the first conflict trial (Conflict trial effect: Non-conflict vs. conflict trial 2: est. = −0.31, SE = 0.14, z-ratio = 2.18, *p* = 0.08; conflict trial 1 vs. conflict trial 2: est. = −0.31, SE = 0.14, z-ratio = 2.15, *p* = 0.08), dogs were significantly faster than wolves overall (Species effect: Dogs vs. wolves: *β* ± SE = −0.93 ± 0.20; *p* < 0.001) (Figure 5A), and the animals approached faster if the human had received the food (Food effect: Animal vs. Human receives food: *β* ± SE = 0.58 ± 0.12; *p* < 0.001).

### 3.4 Conflict-independent species differences

Finally, several variables showed differences between the species, and between whether the animal or the human received the food, regardless of whether a conflict occurred or not. Dogs were more likely to show self-directed behaviors than wolves during the Interaction phase (Species effect: Dogs vs. Wolves: est. = 1.3, SE = 0.54, z-ratio = 2.42, *p* = 0.02; for confidence intervals see Supplementary material), and both showed more self-directed behaviors if the human rather than the animal received the food (Food effect: Animal vs. Human: est. = −1.47, SE = 0.42, z-ratio = −3.53, *p* < 0.001). Contrarily, dogs were less likely than wolves to show self-directed behaviors in the post phase (Species effect: Dogs vs. Wolves: est. = −1.58, SE = 0.34, z-ratio = −4.36, *p* < 0.001) (Figure 3A). Similarly to the latency to approach the human, dogs spent more time than wolves within one body length of the human after the interaction (Species effect: Dogs vs. Wolves: est. = 0.59, SE = 0.19, z-ratio = 3.06, *p* = 0.02) (Figure 5B) and both species spent more time in proximity if the human rather than the animal received the food (Food effect: Animal vs. Human received the food: est. = −0.23, SE = 0.11, z-ratio = −2.19, *p* = 0.03). The same pattern occurred for the proportion of time the animals spent looking at the human during the interaction phase (Species effect: Dogs vs. Wolves): est. = 0.59, SE = 0.16, z-ratio = 3.78, *p* < 0.001; Food effect: Animal vs. Human received the food: est. = −0.67, SE = 0.01, z-ratio = −6.83, *p* < 0.001), and the likelihood to look at the human during the Post phase (Species effect: Dogs vs. Wolves: est. = 1.81, SE = 0.53, z-ratio = 3.45, *p* < 0.001; Food effect: Animal vs. Human: est. = −1.65, SE = 0.54, z-ratio = −3.07, *p* = 0.002). Finally, after the conflict, the Conflict condition effect disappeared and only species influenced tail wagging, with a longer duration in dogs compared to wolves (Species effect: Dogs vs. Wolves: est. = 1.02, SE = 0.16, z-ratio = 6.22, *p* < 0.001).

**Figure 6:**
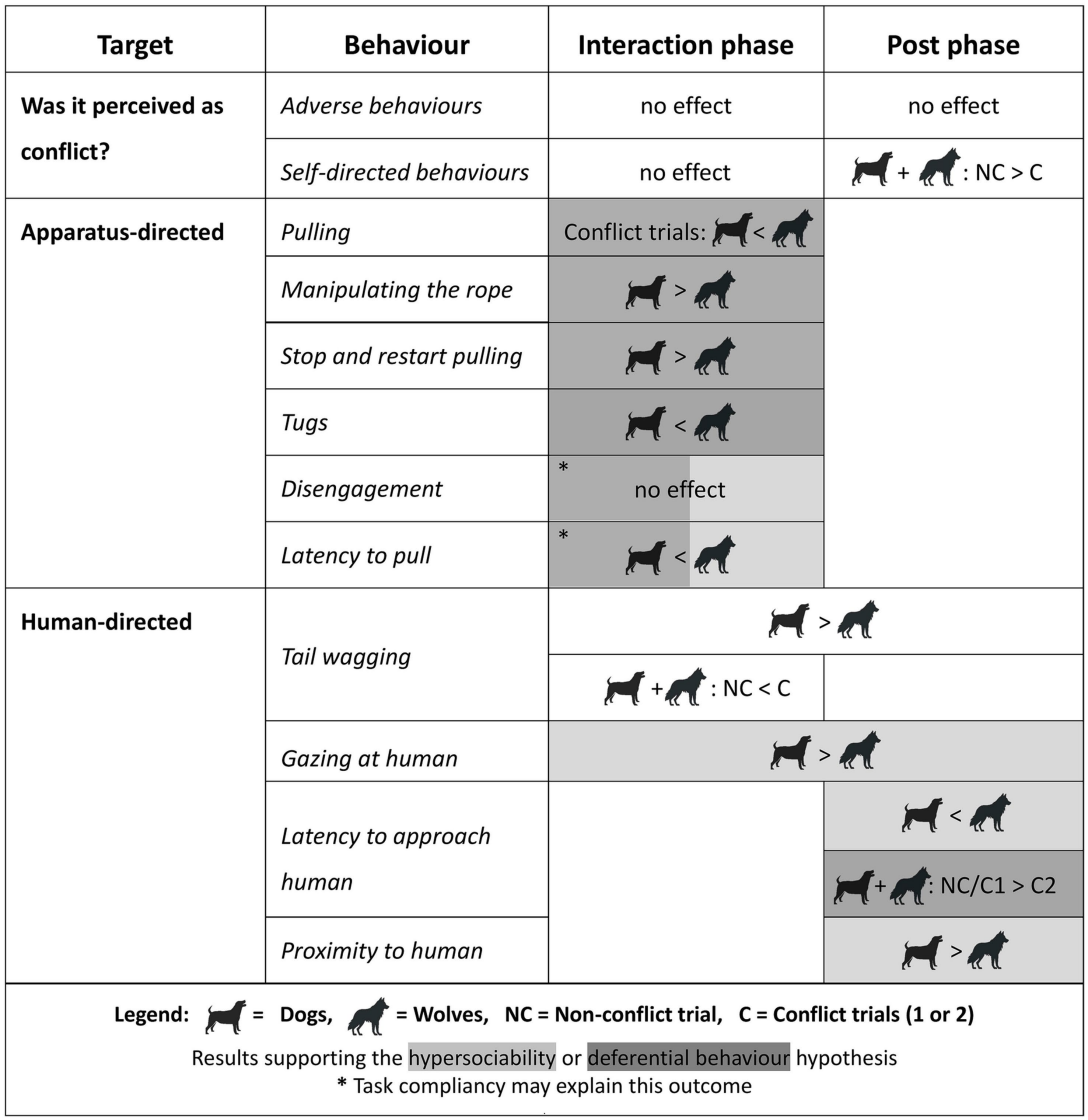
Results summary. The table displays the main results—the effects of Species and Conflict condition—for the different behaviors measured in the Interaction phase and the Post phase. Results consistent with the deferential behavior hypothesis, as predicted by us in Table 2, are highlighted in dark grey, while the results consistent with the hypersociability hypothesis are highlighted in light grey.

We summarize the outcomes in Figure 6.

## 4 Discussion

The hypothesis that dogs’ outstanding human-seeking behavior is driven by an exaggerated sociability (vonHoldt et al., 2017) has been challenged by recent comparative studies, finding increased signals of ambiguity and appeasement in dogs during human interactions (Wirobski et al., 2021; Burkhard et al., 2023; Capitain et al., 2025) and numerous accounts of willingness to accept the humans’ lead compared to wolves (Range et al., 2019; Ujfalussy et al., 2020; Vasconcellos et al., 2016). Instead, it has been proposed that steeper hierarchical relationships (Wynne, 2021; Marshall-Pescini et al., 2017), a selection for deferential behavior (Range et al., 2019; Wirobski et al., 2025) and the corresponding need to please the human might constitute the underlying motivation. Because these hypotheses have been difficult to disentangle in amicable interactions (Lazzaroni et al., 2020b; Wirobski et al., 2021), we created a controlled conflict situation between a bonded human and a dog or wolf to compare their willingness to engage in a conflict over food with the human. However, the results are not fully aligned with either hypothesis. In accordance with the deferential behavior hypothesis, some interaction effects emerged between species and conflict in the engagement behaviors, with wolves pulling significantly longer and more frequently during the conflict than dogs. Against the predictions, however, dogs showed no pronounced signs of disengagement overall. Simultaneously, their human-affiliative behavior remained higher compared to wolves regardless of conflict, as predicted by the hypersociability hypothesis. We interpret the findings in terms of competing compliancy goals and how influential the conflict really was.

The first prerequisite to interpreting the results is to determine whether the animals indeed perceived the setup as a conflict. The self-directed and adverse behaviors present a mixed picture. While the behaviors were not affected by conflict during the interaction, both species were less likely to show self-directed behaviors after the conflict compared to the non-conflict interaction, suggesting that the animals differentiated between the conflict and non-conflict condition. Considering that self-directed behaviors are often interpreted as signals of stress (Hekman et al., 2012), appeasement (Kuhne et al., 2014; Pedretti et al., 2023a), and/or submission (Mech and Boitani, 2003), one would intuitively expect them to increase rather than decrease with conflict. However, recent studies revealed that the communicative role of these behaviors is more nuanced. For example, Firnkes et al. (2017) and Pedretti et al. (2023b) found that self-directed behaviors occur more frequently in (submissive) greeting and ambiguous situations, while higher-threat situations led to a decrease in these behaviors. The lower frequency of self-directed behaviors in our sample after the conflict compared to the non-conflict trials may hence subtly suggest that they perceived the conflict as such. However, Firnkes et al. (2017) also suggested that during threatening encounters, dogs may forego self-directed behaviors in favor of more overt submissive or reactive behaviors to solve the conflict. This was not the case either in our sample, questioning the earlier deduction. Other behaviors that differed between the conflict and non-conflict trials, such as increased tail wagging, shorter latency to approach the human in the second conflict trial, and more pulling at the rope, provide additional but inconclusive evidence. While tail wagging during social approach is shown particularly in more submissive situations (Harrington et al., 2003; Cafazzo et al., 2010), and a shorter latency to approach may be seen as reconciliation (Lazzaroni et al., 2017; De Waal and van Roosmalen, 1979; Walters et al., 2020), neither of these are definitive in isolation. Coincidentally, two recent studies looking at dogs’ reaction towards conflict between two owners used a similar design as ours, including a tug of war with loud protesting (Rial et al., 2025a; Rial et al., 2025b). While the dogs’ stress and consolidation behavior suggested that the dogs understood the situation as a conflict, their setup consisted of a longer and more intense conflict phase than ours (Rial et al., 2025a, Rial et al., 2025b). Since the animals in our study were unaccustomed to conflict with their trainers, we had hoped that a low-level confrontation would be sufficient to trigger deferential behavior while preserving the social bonds and mutual trust that allows for safe, daily hands-on interactions with the animals. However, it is possible that the conflict was too mild to elicit a stronger response. Nevertheless, the subtle differences suggest that the conflict had at least some effect on both species, allowing us to test our hypotheses against the food competition setup. Hence, we do proceed with the interpretation, but with considerable caution.

The second prerequisite for interpreting the results was to ascertain that the differences in the species’ behavior were not merely due to differences in how the humans behaved towards the dogs and wolves (Capitain et al., 2025). Trainers were instructed to behave consistently across all animals, and any deviations were corrected by the experimenter. This was confirmed by the lack of significant species differences in the human behaviors, except for the duration of pulling at the rope, which was longer with wolves. However, our results on the animal differences support the observational conclusion that this was due to the wolves’ more insistent pulling behavior. The trainer did not only have to pull more consistently during the conflict to counter the wolves’ insistence, but they also had to pull for longer whenever the human was supposed to get the food because contrary to the dogs, the wolves refused to let go (more on that below). We hence confidently proceed into the species comparison, disregarding the human biases as causative explanations.

Dogs and wolves most pronouncedly differed in their insistence with which they engaged in the conflict. While there was no species difference in the pulling duration during the non-conflict trials, wolves pulled significantly longer and tucked at the rope more than the dogs during the conflict. As already indicated above, the difference in the human conflict partners’ need to actively pull longer underlines that the dogs were more likely to let go once the humans increased the traction, while this was not the case for wolves. Notably, the strength needed to pull the rope away from the animals was not the reason, given that with the pully system, the human conflict partners could pull the rope towards them easily. However, once the animal was pulled so far that its nose touched the fence, the human had to resort to holding against the rope until the animal let go to prevent injuring the animals. Thinking of previous dog-wolf comparisons, one might suggest that it was not the humans’ arguing that put the dogs off, but rather, that they are generally less persistent than wolves when it comes to problem solving and difficult tasks (Lazzaroni et al., 2020a; Brubaker et al., 2017). However, even in these studies, dogs typically engaged for longer durations than observed here (Miklósi et al., 2003, mean 30s before dogs stopped to look up at the human Lazzaroni et al., 2020a). We had deliberately chosen such a short time for the static tug of war to counteract any issues stemming from differences in task persistence and confidently attribute the differences between the species to the conflict situation rather than the task itself. Likewise, previous studies evidence that both species are adept at reading and cooperating with the human (Heberlein et al., 2016; Range et al., 2019; Udell et al., 2012; Udell et al., 2008; Range and Virányi, 2015), disarming the notion that differences may stem from the wolves’ lacking ability to understand the human’s communication (Hare et al., 2010).

Rather, we suggest that the dogs’ reaction might have been one of uncertainty whether to engage with the trained task or comply with the human’s opposition. This interpretation is supported by the other results. On the one hand, the dogs showed more frequent manipulation (rather than pulling) of the rope, a higher tendency to drop and reinitiate pulling, and an increased gazing at the human during the conflict compared to wolves, suggesting more indecision and monitoring of the human. Likewise, while rare, only dogs—but never wolves—refused to enter the test enclosure for the next trial after experiencing the first conflict. Against our expectations regarding the deferential behavior hypothesis, there was no difference in the likelihood of disengaging from the task during the conflict interaction. On the contrary, dogs were significantly faster than wolves to approach the apparatus and start pulling across all trials, regardless of previous conflict. Perhaps this was due to not knowing what will happen next, since the animals had experienced an equal amount of conflict and non-conflict trials before the second conflict trial started. However, given the repeated training as well as the two to three rewarded trials preceding the conflict during each test session, it might also be conceivable that their inclination to keep engaging with the task despite the conflict could stem from task compliance, i.e., a willingness to please the human (Ujfalussy et al., 2020; Vasconcellos et al., 2016). If that was the case, the implicitly stated command to start the task (the experimenter’s “okay,” followed by the opening shift door), followed by the conflict behavior and arguing from the human might have led to the speedy approach followed by on-and-off engagement with the apparatus. Together, these results suggest that the two species coped differently with the confrontation, with dogs being more affected by the human’s behavior. Simultaneously, it strongly suggests that future experiments aimed at disentangling the hypersociability and deferential hypothesis should not only create a more pertinent conflict than our attempt—an arguably challenging, if not ethically questionable, task when working with relationships reliant on trusting bonds –, but use a conflict that does not stand in opposition to a previously reinforced, implicitly commanded task.

We had added the two-minute Post phase to observe differences in the affiliation pattern in accordance with reconciliation or distance maintenance strategies (Cafazzo et al., 2018; Cavalli et al., 2016). Interestingly, both species approached the human faster after the second conflict trial compared to the first conflict and the non-conflict trial. While this could be interpreted as a trial number effect, past studies showed that the animals generally become slower rather than faster across repeated trials, particularly when they experience reduced or no rewards as it was the case with our human approach (Wilson et al., 2023; Range et al., 2012; Dzik et al., 2024). Alternatively, this could be interpreted as an attempt at reconciliation. Experiencing the second conflict after a conflict just occurred might be perceived as a scary verification that the first trial was not just a fluke, and something was truly off with the relationship. This interpretation might be further reinforced by the fact that reconciliation occurs particularly towards individuals much higher in status (Cafazzo et al., 2018; Cools et al., 2008), and that our animals were not only bonded with the trainer but also relied on them for food (Burkhard et al., 2023). We thereby add to the evidence that while dogs may show a distance-maintenance strategy post conflict in some cases (Cafazzo et al., 2018; Cools et al., 2008; Rial et al., 2025b), reconciliation seems to likewise be part of their post conflict strategy (Walters et al., 2020; Cools et al., 2008; Cavalli et al., 2016). Lastly, one might want to add that the animals’ faster approach, longer gazing, and increased time in proximity of the human after they lost the confrontation—here: by not getting the food—may be evidence for increased reconciliation during more high-stake situations (De Waal and Aureli, 1997; Cordoni and Palagi, 2008). However, we caution that there was no effect of conflict condition. Rather, the animals seemed to follow the human in the expectancy to receive the food, as they usually would in daily life.

Finally, there are a few behaviors left to discuss, all of which pertained to the dogs showing more affiliation towards the human than the wolves, regardless of previous conflict: More tail wagging, gazing, time spent in proximity, and a faster approach towards the human after the Interaction phase. Following the deferential behavior hypothesis, this is contrary to our assumption that conflict experience would alter dogs’ proximity-seeking. Again, the same two possible interpretation-approaches as above come into play. One the one hand, this outcome supports what we predicted under the hypersociability hypothesis—exaggerated sociability despite the human’s negative reaction. Interestingly, the William Beuren Syndrome in humans, the hypersocial genetic condition underpinned by the same genetic variation dogs are thought to have been selected for (vonHoldt et al., 2010; vonHoldt et al., 2017), is associated with difficulty reading and hence appropriately reacting to others’ negative affect (Järvinen et al., 2013; Santos et al., 2010). One could hence argue that the same cause might be underlying dogs’ persistent affiliation in our study. However, dogs’ ability to appropriately react to negative human expressions is well evidenced (Lazzaroni et al., 2023; Albuquerque et al., 2016; Albuquerque et al., 2022; Barber et al., 2017; Siniscalchi et al., 2018a,b; Rial et al., 2025b). On the other hand, as discussed with the latency above, it is also possible that the conflict trials were too few to have a lasting impact, or that they saw it as a confrontation that did not extend beyond the apparatus, hence approaching the human despite it. As above, this cannot yet be answered without a more rigorous confrontation.

We appreciate that the relatively small sample size impedes generalization, as does the unique rearing history of the study subjects. Consequently, subtle species differences may have gone undetected, particularly if the conflict manipulation elicited relatively weak behavioral divergence. Future studies with larger samples may help provide more precise effect size estimates and clarify whether the observed effects reflect genuinely small species differences or limited statistical power. At the same time, we point out that the animals were extensively socialized with both humans and conspecifics, providing a well-rounded life experience that allows for conspecific and heterospecific behavioral patterns to emerge. In addition, the similar raising and keeping of both species in conspecific packs with daily human interaction allows us to compare species-intrinsic behavior rather than environmental confounds. While we concede that factors such as reinforcement history (i.e., if the animal received food in the previous trial) may still have taken effect, we point out that the fact that we tested all available animals, counterbalanced reinforcement across trials and subjects, and employed a repeated test design to increase power limits such confounders as much as possible given the sample size without risking habituation effects.

Taken together, our findings suggest that our conflict setup may not have been ideally suited in strength and context to fully tease dogs’ human approach behavior apart in terms of the hypersociability and deferential behavior hypotheses. On the one hand, the dogs did show a less fierce engagement in the confrontation compared to the wolves, as well as more sustained human proximity. This underlines that although dogs and wolves remain closely related to a point where they are biologically classified as the same species, domestication may have involved strong selection on behavioral traits that may have produced meaningful differences in social behavior and responses to conflict situations despite the relatively recent evolutionary divergence. On the other hand, dogs were consistent in their engagement across trials, contrary to our predictions underlying the deferential hypothesis. While the consistency might partially stem from the few repetitions and thus few possibilities to understand the conflict as such, the combined results also point to the interpretation that dogs’ willingness to comply with the previously trained, requested task might have competed with tractability in response to the human’s adverse reaction to their engagement. A conflict that is not based on a previously human-reinforced task will be needed to verify this interpretation. Similarly, the interpretation of whether the sustained human proximity-seeking in dogs was present despite conflict, supporting the hypersociability hypothesis, or remained because the dogs did not perceive it as an influential conflict, remains an open one. Nevertheless, controlling for the human behavior not only in theory but by specifically analyzing it gives us certainty that the differences we saw in the animals’ behavior were actual species differences, not the human partners’ biases, providing a solid foundation for further exploration of these hypotheses.

## Data Availability

The datasets in this study can be found in the supplement as well as in online repositories with the following doi: 10.6084/m9.figshare.31961016.
