# Supplementary material for: Deference or sociability? Insights from dogs’ and wolves’ human-directed behavior in a food-conflict task
Source: Front Psychol. 2026 Jun 30;17:1854682. doi: 10.3389/fpsyg.2026.1854682 (PMC13365327; doi:10.3389/fpsyg.2026.1854682)
Supplement: Supplementary file 2 [file Table_2.DOCX]

Supplementary Material

**Deference or Sociability? Insights from Dogs’ and Wolves’ Human-directed Behaviour in a Food-Conflict Task**

Authors

Svenja Capitain^1*^, Sarah Marshall-Pescini^1^, Gwendolyn Wirobski^1,2^, Tabea Teichmann^1^ & Friederike Range^1^

^1^ Domestication Lab, Konrad Lorenz Institute of Ethology, University of Veterinary Medicine Vienna, 1210 Vienna, Austria

² Comparative Cognition Group, Institute of Biology, Faculty of Science, Université de Neuchâtel, 2000 Neuchâtel, Switzerland

Corresponding Author: [Svenja.capitain@vetmeduni.ac.at](mailto:Svenja.capitain@vetmeduni.ac.at), [Friederike.range@vetmeduni.ac.at](mailto:Friederike.range@vetmeduni.ac.at)

Content

[**1.** **INTERRATER RELIABILITY – Animals** 2](#_Toc226555645)

[**2.** **INTERRATER RELIABILITY – Humans** 2](#_Toc226555646)

[**3.** **STATISTICAL ANALYSIS – Humans** 3](#_Toc226555647)

[**4.** **STATISTICAL ANALYSIS – Animals – Interaction Phase** 7](#_Toc226555648)

[**5.** **STATISTICAL ANALYSIS – Animals – Post Phase** 18](#_Toc226555649)

## **INTERRATER RELIABILITY – Animals**

To assess the interrater reliability of the animals’ behaviour, 20% (22 videos) were re-coded by a second coder and the outcomes were compared through an interclass correlation coefficient (ICC).

| **Category** | **Behaviour** | **Reliability (ICC)** |
| --- | --- | --- |
| **Engagement** | Engagement | 1.0 |
|  | Pulling | 0.83 |
|  | Pulls | 0.97 |
|  | Manipulating apparatus | 0.94 |
|  | Latency to get in proximity | 0.90 |
|  | Latency to start pulling | 0.96 |
|  | Proximity to the apparatus | 0.96 |
|  | | |
| **Human-directed** | Gazing towards conflict partner | 0.67 |
|  | Tail wagging | 0.99 |
|  | Latency to approach human | 0.95 |
|  | Proximity to human | 0.96 |
|  | | |
| **Conflict-indicators** | Adverse | 0.98 |
|  | Self-directed | 0.87 |
|  | | |
| **Average ICC** | | **0.92** |

## **INTERRATER RELIABILITY – Humans**

To assess the interrater reliability of the human partners’ behaviour, 20% (22 videos) were re-coded by a second coder, blind to the hypotheses and the interacting animal, and the outcomes were compared through an interclass correlation coefficient (ICC).

| **Behaviour** | **Reliability (ICC)** |
| --- | --- |
| Gazing at the animal (D) | 0.85 |
| Leaning forward (D) | 0.98 |
| Pulling actively (D) | 0.97 |
| Talking to the animal (D) | 0.83 |
| Use of one hand to pull (D) | 0.95 |
| Voice aggressiveness (D) | 0.84 |
| **Average ICC** | **0.90** |

## **STATISTICAL ANALYSIS – Humans**

**Duration gazing at the animal**

full.LookD <- glmmTMB(All_During_Looking.at.animal__Total.duration ~ Species + Trial.z , family = beta_family, data = LookD)

**Full-null model comparison:**

|  | Df | AIC | BIC | logLik | deviance | Chisq Chi | Df | Pr(>Chisq) |  |
| --- | --- | --- | --- | --- | --- | --- | --- | --- | --- |
| null.LookD | 3.00 | -518.23 | -510.27 | 262.12 | -524.23 |  |  |  |  |
| full.LookD | 4.00 | -518.52 | -507.90 | 263.26 | -526.52 | 2.29 | 1.00 | 0.13 |  |

**Model summary:**

|  | Estimate | Std. Error | z-value | Pr(>\|z\|) |  |
| --- | --- | --- | --- | --- | --- |
| (Intercept) | 3.05 | 0.16 | 18.87 | <2e-16 | *** |
| SpeciesWolf | 0.28 | 0.18 | 1.51 | 0.13 |  |
| Trial.z | 0.02 | 0.09 | 0.20 | 0.84 |  |

**Confidence intervals:**

|  | orig | X2.5. | X97.5. |
| --- | --- | --- | --- |
| cond@(Intercept) | 3.05 | 2.74 | 3.38 |
| cond@SpeciesWolf | 0.28 | -0.07 | 0.65 |
| cond@Trial.z | 0.02 | -0.17 | 0.20 |
| disp@(Intercept) | 2.44 | 2.15 | 2.83 |

**Duration talking**

full.Talk <- glmmTMB(All_talking__Total.duration ~ Species + Trial.z + (1+Trial.z||AnimalID) + (1+Trial.z||HumanID), family = beta_family, data = Talk)

**Full-null model comparison:**

|  | Df | AIC | BIC | logLik | deviance | Chisq Chi | Df | Pr(>Chisq) |  |
| --- | --- | --- | --- | --- | --- | --- | --- | --- | --- |
| null.Talk | 7.00 |  |  |  |  |  |  |  |  |
| full.Talk | 8.00 | -151.03 | -129.80 | 83.52 | -167.03 | 0.00 | 1.00 | 1.00 |  |

**Model summary:**

|  | Estimate | Std. Error | z-value | Pr(>\|z\|) |  |
| --- | --- | --- | --- | --- | --- |
| (Intercept) | 1.43 | 0.22 | 6.38 | 0.00 | *** |
| SpeciesWolf | -0.48 | 0.30 | -1.59 | 0.11 |  |
| Trial.z | 0.13 | 0.05 | 2.40 | 0.02 | * |

**Confidence intervals:**

|  | orig | X2.5. | X97.5. |
| --- | --- | --- | --- |
| cond@(Intercept) | 1.43 | 0.99 | 1.91 |
| cond@SpeciesWolf | -0.48 | -1.06 | 0.08 |
| cond@Trial.z | 0.13 | 0.02 | 0.24 |
| disp@(Intercept) | 2.92 | 2.66 | 3.32 |

**Duration leaning forward**

full.Forward <- glmmTMB(All_forward.mean__Total.duration ~ Species + Trial.z + (1+Trial.z||AnimalID) + (1+Trial.z||HumanID), family = beta_family, data = Forward)

**Full-null model comparison:**

|  | Df | AIC | BIC | logLik | deviance | Chisq Chi | Df | Pr(>Chisq) |  |
| --- | --- | --- | --- | --- | --- | --- | --- | --- | --- |
| null.Forward | 7.00 | -89.45 | -70.87 | 51.73 | -103.45 |  |  |  |  |
| full.Forward | 8.00 | -87.69 | -66.45 | 51.84 | -103.69 | 0.24 | 1.00 | 0.63 |  |

**Model summary:**

|  | Estimate | Std. Error | z-value | Pr(>\|z\|) |  |
| --- | --- | --- | --- | --- | --- |
| (Intercept) | -1.20 | 0.18 | -6.63 | 0.00 | *** |
| SpeciesWolf | 0.12 | 0.25 | 0.49 | 0.63 |  |
| Trial.z | -0.01 | 0.10 | -0.14 | 0.89 |  |

|  | orig | X2.5. | X97.5. |
| --- | --- | --- | --- |
| cond@(Intercept) | -1.20 | -1.57 | -0.85 |
| cond@SpeciesWolf | 0.12 | -0.34 | 0.60 |
| cond@Trial.z | -0.01 | -0.22 | 0.19 |
| disp@(Intercept) | 1.92 | 1.60 | 2.30 |

**Confidence intervals:**

**Duration holding the rope in two hands**

full.Hands2 <- glmmTMB(All_Hands_2hands_Total.duration ~ Species + Trial.z + (1+Trial.z||AnimalID), family = beta_family, data = Hands2)

**Full-null model comparison:**

|  | Df | AIC | BIC | logLik | deviance | Chisq Chi | Fd | Pr(>Chisq) |  |
| --- | --- | --- | --- | --- | --- | --- | --- | --- | --- |
| null.Hands2 | 5.00 | -233.62 | -220.3 | 121.81 | -243.62 |  |  |  |  |
| full.Hands2 | 6.00 | -231.62 | -215.7 | 121.81 | -243.62 | 0.00 | 1.00 | 0.97 |  |

**Model summary:**

|  | Estimate | Std. Error | z-value | Pr(>\|z\|) |  |
| --- | --- | --- | --- | --- | --- |
| (Intercept) | -1.44 | 0.51 | -2.85 | 0.00 | ** |
| SpeciesWolf | -0.03 | 0.69 | -0.04 | 0.96 |  |
| Trial.z | 0.00 | 0.09 | 0.02 | 0.98 |  |

|  | orig | X2.5. | X97.5. |
| --- | --- | --- | --- |
| cond@(Intercept) | -1.44 | -2.49 | -0.50 |
| cond@SpeciesWolf | -0.03 | -1.42 | 1.23 |
| cond@Trial.z | 0.00 | -0.17 | 0.19 |
| disp@(Intercept) | 1.51 | 1.24 | 1.93 |

**Confidence intervals:**

**Voice aggressiveness**

full.VAgg <- glmmTMB(All_Voice.aggressiveness_calced_Total.duration ~ Species + Trial.z + (1+Trial.z||AnimalID), family = beta_family, data = VAgg)

**Full-null model comparison:**

|  | Df | AIC | BIC | logLik | deviance | Chisq Chi | Df | Pr(>Chisq) |  |
| --- | --- | --- | --- | --- | --- | --- | --- | --- | --- |
| null.VAgg | 5.00 | -1253.4 | -1240.2 | 631.72 | -1263.4 |  |  |  |  |
| full.VAgg | 6.00 | -1251.4 | -1235.5 | 631.72 | -1263.4 | 0.00 | 1.00 | 0.97 |  |

**Model summary:**

|  | Estimate | Std. Error | z-value | Pr(>\|z\|) |  |
| --- | --- | --- | --- | --- | --- |
| (Intercept) | 4.40 | 0.25 | 17.53 | <2e-16 | *** |
| SpeciesWolf | 0.01 | 0.19 | 0.04 | 0.97 |  |
| Trial.z | 0.04 | 0.10 | 0.44 | 0.66 |  |

**Confidence intervals:**

|  | orig | X2.5. | X97.5. |
| --- | --- | --- | --- |
| cond@(Intercept) | 4.40 | 3.98 | 4.99 |
| cond@SpeciesWolf | 0.01 | -0.38 | 0.39 |
| cond@Trial.z | 0.04 | -0.14 | 0.22 |
| disp@(Intercept) | 2.61 | 2.25 | 3.26 |

**Duration actively pulling the rope**

full.Apull <- glmmTMB(All_Active.pulling__Total.duration ~ Species + Trial.z + (1+Trial.z||AnimalID) + (1+Trial.z||HumanID), family = beta_family, data = Apull)

**Full-null model comparison:**

|  | Df | AIC | BIC | logLik | deviance | Chisq Chi | Df | Pr(>Chisq) |  |
| --- | --- | --- | --- | --- | --- | --- | --- | --- | --- |
| null.Apull | 7 | -96.79 | -78.22 | 55.40 | -110.79 |  |  |  |  |
| full.Apull | 8 | -101.58 | -80.35 | 58.79 | -117.58 | 6.78 | 1.00 | 0.01 | ** |

**Model summary:**

|  | Estimate | Std. Error | z-value | Pr(>\|z\|) |  |
| --- | --- | --- | --- | --- | --- |
| (Intercept) | -1.42 | 0.17 | -8.27 | <0.001 | *** |
| SpeciesWolf | 0.64 | 0.23 | 2.76 | 0.01 | ** |
| Trial.z | 0.11 | 0.07 | 1.45 | 0.15 |  |

**Confidence intervals:**

|  | orig | X2.5. | X97.5. |
| --- | --- | --- | --- |
| cond@(Intercept) | -1.42 | -1.80 | -1.07 |
| cond@SpeciesWolf | 0.64 | 0.19 | 1.12 |
| cond@Trial.z | 0.11 | -0.03 | 0.26 |
| disp@(Intercept) | 2.08 | 1.83 | 2.46 |

**Post-hoc:**

| contrast | estimate | SE | df | z.ratio | p.value |
| --- | --- | --- | --- | --- | --- |
| Dog - Wolf | -0.64 | 0.23 | Inf | -2.76 | 0.006 |

## **STATISTICAL ANALYSIS – Animals – Interaction Phase**

**Self-directed behaviours**

C_model_Displ <- glmmTMB(Pre.Displacement.Binary ~ Species * Phase + Sex + Food + Session + Age.z + offset(log(Pre_Prox_ManPull))+ (1 + Food.H + Session.z||AnimalID), data = Displ.data, family = binomial(link = "logit"))

**Full-null model comparison:**

|  | Df | AIC | BIC | logLik | deviance | Chisq Chi | Df | Pr(>Chisq) |
| --- | --- | --- | --- | --- | --- | --- | --- | --- |
| C_model_Displ.0 | 8 | 331.38 | 360.52 | -157.69 | 315.38 |  |  |  |
| C_model_Displ | 11 | 328.4 | 368.46 | -153.2 | 306.4 | 8.98 | 3 | 0.03 |

|  | Estimate | Std. Error | z-value | Pr(>\|z\|) |  |
| --- | --- | --- | --- | --- | --- |
| (Intercept) | -3.07 | 0.67 | -4.61 | 0.00 | *** |
| SpeciesWolf | -1.43 | 0.58 | -2.48 | 0.01 | * |
| PhaseNC | -0.77 | 0.63 | -1.23 | 0.22 |  |
| SexW | -0.09 | 0.51 | -0.18 | 0.86 |  |
| FoodH | 1.43 | 0.44 | 3.28 | 0.00 | ** |
| Session | 0.17 | 0.15 | 1.12 | 0.26 |  |
| Age.z | 0.04 | 0.28 | 0.14 | 0.89 |  |
| SpeciesWolf:PhaseNC | -0.01 | 0.95 | -0.01 | 1.00 |  |

**Model summary:**

**Reduced model summary:**

|  | Estimate | Std. Error | z-value | Pr(>\|z\|) |  |
| --- | --- | --- | --- | --- | --- |
| (Intercept) | -3.07 | 0.66 | -4.63 | 0.00 | *** |
| SpeciesWolf | -1.43 | 0.56 | -2.55 | 0.01 | * |
| PhaseNC | -0.78 | 0.49 | -1.58 | 0.11 |  |
| SexW | -0.09 | 0.51 | -0.18 | 0.86 |  |
| FoodH | 1.43 | 0.44 | 3.28 | 0.00 | ** |
| Session | 0.17 | 0.15 | 1.12 | 0.26 |  |
| Age.z | 0.04 | 0.28 | 0.14 | 0.89 |  |

**Posthoc:**

| contrast | estimate | SE | df | z.ratio | p.value |  |  |
| --- | --- | --- | --- | --- | --- | --- | --- |
| Dog - Wolf | 1.43 | 0.561 | Inf | 2.553 | 0.0107 |  |  |
| A-H | -1.43 | 0.438 | Inf | -3.277 | 0.001 |  |  |
|  |  |  |  |  |  |  |  |
|  |  |  |  |  |  |  |  |

**Confidence intervals:**

|  | orig | X2.5. | X97.5. |
| --- | --- | --- | --- |
| cond@(Intercept) | -3.07 | -4.55 | -2.03 |
| cond@SpeciesWolf | -1.43 | -2.76 | -0.25 |
| cond@PhaseNC | -0.77 | -2.29 | 0.51 |
| cond@SexW | -0.09 | -0.98 | 0.78 |
| cond@FoodH | 1.43 | 0.78 | 2.42 |
| cond@Session | 0.17 | -0.14 | 0.55 |
| cond@Age.z | 0.04 | -0.48 | 0.77 |
| cond@SpeciesWolf:PhaseNC | -0.01 | -17.48 | 1.91 |

**Adverse behaviours** (Full-null model: χ²=3.11, df=8, *p*=0.37)

C_model_Avers.n.D <- glmmTMB(Adverse.NonDispl.Binary ~ Species * Phase + Sex + Food + Session + Age.z + (1 + Food.H + Session.z || AnimalID) + offset(log(Pre_Prox_ManiPull)), data = Adv.nD.data, family = binomial(link = "logit"))

**Full-null model comparison:**

|  | Df | AIC | BIC | logLik | deviance | Chisq Chi | Df | P-value |
| --- | --- | --- | --- | --- | --- | --- | --- | --- |
| C_model_Avers.n.D.0 | 8 | 126.87 | 156.15 | -55.44 | 110.87 |  |  |  |
| C_model_Avers.n.D | 11 | 130.29 | 170.55 | -54.15 | 108.29 | 2.58 | 3 | 0.46 |

**Model summary:**

|  | Estimate | Std. Error | z-value | Pr(>\|z\|) |  |
| --- | --- | --- | --- | --- | --- |
| (Intercept) | -5.81 | 1.53 | -3.79 | 0.00 | *** |
| SpeciesWolf | 0.50 | 1.25 | 0.40 | 0.69 |  |
| PhaseNC | -19.06 | 14020.91 | 0.00 | 1.00 |  |
| SexW | 0.57 | 1.15 | 0.49 | 0.62 |  |
| FoodH | -0.18 | 0.65 | -0.28 | 0.78 |  |
| Session | -0.38 | 0.31 | -1.22 | 0.22 |  |
| Age.z | 1.73 | 0.81 | 2.14 | 0.03 | * |
| SpeciesWolf:PhaseNC | 18.13 | 14020.91 | 0.00 | 1.00 |  |

**Confidence intervals:**

|  | orig | X2.5. | X97.5. |
| --- | --- | --- | --- |
| cond@(Intercept) | -5.81 | -36.11 | -3.34 |
| cond@SpeciesWolf | 0.50 | -2.67 | 26.51 |
| cond@PhaseNC | -19.06 | -59.44 | -3.72 |
| cond@SexW | 0.57 | -3.14 | 2.87 |
| cond@FoodH | -0.18 | -2.16 | 1.28 |
| cond@Session | -0.38 | -1.07 | 0.24 |
| cond@Age.z | 1.73 | 0.71 | 4.92 |
| cond@SpeciesWolf:PhaseNC | 18.12 | -9.28 | 51.51 |

**Duration pulling**

C_model_Pull <- glmmTMB(Pre_Pulling_PropP ~ Species * Trial + Sex + Session + Age.z + (1 + Session.z||AnimalID) , data = Pulling.data, family=beta_family)

**Full-null model comparison:**

|  | Df | AIC | BIC | logLik | deviance | Chisq Chi | Df | Pr(>Chisq) |  |
| --- | --- | --- | --- | --- | --- | --- | --- | --- | --- |
| C_model_PullP.0 | 7 | -292.88 | -267.36 | 153.44 | -306.88 |  |  |  |  |
| C_model_PullP | 10 | -394.24 | -357.79 | 207.12 | -414.24 | 107.36 | 3 | <0.001 | *** |

**Model summary:**

|  | Estimate | Std. Error | z-value | Pr(>\|z\|) |  |
| --- | --- | --- | --- | --- | --- |
| (Intercept) | -1.80 | 0.20 | -8.86 | <0.001 | *** |
| SpeciesWolf | 0.98 | 0.20 | 4.85 | 0.00 | *** |
| PhaseNC | -0.79 | 0.17 | -4.54 | 0.00 | *** |
| SexW | 0.10 | 0.19 | 0.55 | 0.58 |  |
| Session | 0.10 | 0.04 | 2.75 | 0.01 | ** |
| Age.z | -0.26 | 0.10 | -2.52 | 0.01 | * |
| SpeciesWolf:PhaseNC | -0.47 | 0.23 | -2.09 | 0.04 | * |

**Confidence intervals:**

|  | orig | X2.5. | X97.5. |
| --- | --- | --- | --- |
| cond@(Intercept) | -1.80 | -2.21 | -1.40 |
| cond@SpeciesWolf | 0.98 | 0.59 | 1.39 |
| cond@PhaseNC | -0.79 | -1.07 | -0.47 |
| cond@SexW | 0.10 | -0.27 | 0.48 |
| cond@Session | 0.10 | 0.03 | 0.17 |
| cond@Age.z | -0.26 | -0.47 | -0.06 |
| cond@SpeciesWolf:PhaseNC | -0.47 | -0.91 | -0.09 |
| disp@(Intercept) | 2.45 | 2.29 | 2.64 |

**Post-hoc:**

| contrast | estimate | SE | df | z.ratio | p.value |
| --- | --- | --- | --- | --- | --- |
| Dog C - Wolf C | -0.98 | 0.20 | Inf | -4.85 | <.0001 |
| Dog C - Dog NC | 0.79 | 0.17 | Inf | 4.54 | <.0001 |
| Dog C - Wolf NC | 0.29 | 0.24 | Inf | 1.21 | 0.62 |
| Wolf C - Dog NC | 1.76 | 0.25 | Inf | 7.04 | <.0001 |
| Wolf C - Wolf NC | 1.26 | 0.15 | Inf | 8.49 | <.0001 |
| Dog NC - Wolf NC | -0.50 | 0.28 | Inf | -1.80 | 0.27 |

**Duration manipulating**

C_model_ManipPull <- glmmTMB(Pre_Manipulatin_PropP_Total.Duration ~ Species * Phase + Sex + Session + Age.z + (1 + Session.z||AnimalID) , data = Manip.data, family=beta_family)

**Full-null model comparison:**

|  | Df | AIC | BIC | logLik | deviance | Chisq Chi | Df | Pr(>Chisq) |  |
| --- | --- | --- | --- | --- | --- | --- | --- | --- | --- |
| C_model_ManipPull.0 | 7 | -981.29 | -955.78 | 497.65 | -995.29 |  |  |  |  |
| C_model_ManipPull | 10 | -996.62 | -960.17 | 508.31 | -1016.62 | 21.33 | 3 | 0.00 | *** |

**Model summary:**

|  | Estimate | Std. Error | z-value | Pr(>\|z\|) |  |
| --- | --- | --- | --- | --- | --- |
| (Intercept) | -1.82 | 0.20 | -8.87 | < 0.001 | *** |
| SpeciesWolf | -0.85 | 0.16 | -5.33 | 0.00 | *** |
| PhaseNC | -0.06 | 0.20 | -0.28 | 0.78 |  |
| SexW | 0.21 | 0.13 | 1.57 | 0.12 |  |
| Session | -0.06 | 0.05 | -1.08 | 0.28 |  |
| Age.z | 0.31 | 0.08 | 4.10 | 0.00 | *** |
| SpeciesWolf:PhaseNC | -0.01 | 0.29 | -0.02 | 0.98 |  |

|  | orig | X2.5. | X97.5. |
| --- | --- | --- | --- |
| cond@(Intercept) | -1.82 | -2.24 | -1.41 |
| cond@SpeciesWolf | -0.86 | -1.11 | -0.59 |
| cond@PhaseNC | -0.06 | -0.34 | 0.20 |
| cond@SexW | 0.21 | -0.01 | 0.48 |
| cond@Session | -0.06 | -0.16 | 0.04 |
| cond@Age.z | 0.31 | 0.14 | 0.43 |
| cond@SpeciesWolf:PhaseNC | 1.73 | 1.54 | 1.96 |

**Confidence intervals:**

**Post-hoc:**

| contrast | estimate | SE | df | z.ratio | p.value |
| --- | --- | --- | --- | --- | --- |
| D-W | 0.86 | 0.15 | Inf | 5.711 | <.0001 |

**Frequency restart pulling**

C_model_PullStart <- glmmTMB(Pre_PullingStarts_Freq ~ Species * Phase + Sex + Session + Age.z + (1+Session.z||AnimalID) + offset(log(Pre_Proximity.to.the.apparatus__Total.duration)), data = PullStart.data, family=nbinom2)

**Full-null model comparison:**

|  | Df | AIC | BIC | logLik | deviance | Chisq Chi | Df | Pr(>Chisq) |
| --- | --- | --- | --- | --- | --- | --- | --- | --- |
| C_model_PullStart.0 | 7 | 1256.6 | 1282.4 | -621.32 | 1242.6 |  |  |  |
| C_model_PullStart | 10 | 1179.8 | 1216.6 | -579.88 | 1159.8 | 82.866 | 3 | <0.001 |

**Model summary:**

|  | DF | AIC | LRT | Pr(>Chi) |  |
| --- | --- | --- | --- | --- | --- |
| <none> | 1010.3 |  |  |  |  |
| Sex | 1 | 1018.40 | 10.08 | 0.00 | ** |
| Session | 1 | 1011.00 | 2.71 | 0.10 | . |
| Age.z | 1 | 1008.70 | 0.38 | 0.54 |  |
| Species:Phase | 1 | 1011.10 | 2.75 | 0.10 | . |

|  | Estimate | Std. Error | z-value | p-value |  |
| --- | --- | --- | --- | --- | --- |
| (Intercept) | 0.44 | 0.23 | 1.93 | 0.05 | . |
| SpeciesWolf | -1.21 | 0.20 | -6.13 | 0.00 | *** |
| PhaseNC | 1.21 | 0.15 | 8.16 | 0.00 | *** |
| SexW | -0.68 | 0.19 | -3.57 | 0.00 | *** |
| Session | -0.08 | 0.05 | -1.43 | 0.15 |  |
| Age.z | 0.04 | 0.10 | 0.41 | 0.68 |  |

**Reduced model summary:**

**Posthoc:**

| contrast | estimate | SE | df | z.ratio | p.value |
| --- | --- | --- | --- | --- | --- |
| Dog - Wolf | 1.16 | 0.18 | Inf | 6.477 | <.0001 |
| C - NC | -1.13 | 0.14 | Inf | -8.122 | <.0001 |

**Confidence intervals:**

|  | orig | X2.5. | X97.5. |
| --- | --- | --- | --- |
| cond@(Intercept) | 0.44 | 0.11 | 0.81 |
| cond@SpeciesWolf | -1.21 | -1.58 | -0.78 |
| cond@PhaseNC | 1.21 | 0.93 | 1.45 |
| cond@SexW | -0.68 | -1.06 | -0.31 |
| cond@Session | -0.08 | -0.18 | 0.02 |
| cond@Age.z | 0.04 | -0.12 | 0.21 |

**Post-hoc:**

| contrast | estimate | SE | df | z.ratio | p.value |
| --- | --- | --- | --- | --- | --- |
| Dog C - Wolf C | 2.41 | 0.38 | Inf | 6.34 | <.0001 |
| Dog C - Dog NC | -1.23 | 0.40 | Inf | -3.09 | 0.01 |
| Dog C - Wolf NC | -0.11 | 0.45 | Inf | -0.24 | 0.99 |
| Wolf C - Dog NC | -3.64 | 0.48 | Inf | -7.52 | <.0001 |
| Wolf C - Wolf NC | -2.52 | 0.45 | Inf | -5.62 | <.0001 |
| Dog NC - Wolf NC | 1.12 | 0.54 | Inf | 2.09 | 0.16 |

**Frequency Rope Tucks**

C_model_Pulls <- glmmTMB(Pre_Pulls__Number.of.occurrences ~ Species * Trial + Sex + Session + Age.z + (1 + Session.z||AnimalID) + offset(log(Pre_Pulling__Total.duration)), data = Pulls.data, family=poisson)

**Full-null model comparison:**

|  | Df | AIC | BIC | logLik | deviance | Chisq Chi | Df | Pr(>Chisq) |
| --- | --- | --- | --- | --- | --- | --- | --- | --- |
| C_model_Pulls.0 | 6 | 591.87 | 612.6 | -289.94 | 579.87 |  |  |  |
| C_model_Pulls | 9 | 588.9 | 620 | -285.45 | 570.9 | 8.97 | 3 | 0.03 |

**Model summary:**

|  | Estimate | Std. Error | z-value | Pr(>\|z\|) |  |
| --- | --- | --- | --- | --- | --- |
| (Intercept) | -1.89 | 0.38 | -4.92 | 0.00 | *** |
| SpeciesWolf | 0.88 | 0.37 | 2.39 | 0.02 | * |
| TrialC2 | -0.01 | 0.34 | -0.04 | 0.97 |  |
| SexW | 0.62 | 0.29 | 2.09 | 0.04 | * |
| Session | -0.15 | 0.05 | -2.86 | 0.00 | ** |
| Age.z | 0.29 | 0.15 | 1.90 | 0.06 | . |
| SpeciesWolf:TrialC2 | 0.15 | 0.38 | 0.40 | 0.69 |  |

**Reduced model summary:**

|  | Estimate | Std. Error | z-value | Pr(>\|z\|) |  |
| --- | --- | --- | --- | --- | --- |
| (Intercept) | -1.95 | 0.36 | -5.46 | 0.00 | *** |
| SpeciesWolf | 0.96 | 0.32 | 3.03 | 0.00 | ** |
| TrialC2 | 0.11 | 0.15 | 0.73 | 0.46 |  |
| SexW | 0.61 | 0.29 | 2.09 | 0.04 | * |
| Session | -0.15 | 0.05 | -2.86 | 0.00 | ** |
| Age.z | 0.29 | 0.15 | 1.90 | 0.06 | . |

**Posthoc:**

| contrast | estimate | SE | df | z.ratio | p.value |
| --- | --- | --- | --- | --- | --- |
| Dog - Wolf | 0.958 | 0.316 | Inf | -3.034 | 0.0024 |
| M - W | -0.615 | 0.294 | Inf | -2.092 | 0.0365 |

**Confidence intervals:**

|  | orig | X2.5. | X97.5. |
| --- | --- | --- | --- |
| cond@(Intercept) | -1.95 | -2.74 | -1.35 |
| cond@SpeciesWolf | 0.96 | 0.33 | 1.60 |
| cond@TrialC2 | 0.11 | -0.18 | 0.40 |
| cond@SexW | 0.61 | 0.02 | 1.26 |
| cond@Session | -0.15 | -0.26 | -0.06 |
| cond@Age.z | 0.29 | -0.01 | 0.58 |

**Likelihood disengagement**

C_model_Engage.coll <- glmmTMB(Engagement.Binary.coll ~ Species * Phase + Sex + Age.z + Session.z + (1 |AnimalID), data = Eng.data.coll, family = binomial(link = "logit"))

**Full-null model comparison:**

|  | Df | AIC | BIC | logLik | deviance | Chisq Chi | Df | Pr(>Chisq) |  |
| --- | --- | --- | --- | --- | --- | --- | --- | --- | --- |
| C_model_Engage.coll.0 | 5 | 124.75 | 141.94 | -57.373 | 114.746 |  |  |  |  |
| C_model_Engage.coll | 8 | 97.55 | 125.06 | -40.78 | 81.551 | 33.196 | 3 | >0.001 | *** |

**Model summary:**

|  | Estimate | Std. Error | z-value | Pr(>\|z\|) |
| --- | --- | --- | --- | --- |
| (Intercept) | -1.34 | 1.07 | -1.26 | 0.21 |
| SpeciesWolf | -1.88 | 1.35 | -1.39 | 0.17 |
| PhaseNC | -13.95 | 178.29 | -0.08 | 0.94 |
| SexW | -1.14 | 1.14 | -1.00 | 0.32 |
| Age.z | 0.11 | 0.68 | 0.16 | 0.87 |
| Session.z | 0.14 | 0.32 | 0.45 | 0.65 |
| SpeciesWolf:PhaseNC | -3 | 1443.50 | 0.00 | 1.00 |

**Confidence intervals:**

|  | orig | X2.5. | X97.5. |
| --- | --- | --- | --- |
| cond@(Intercept) | -1.34 | -5.73 | 0.50 |
| cond@SpeciesWolf | -1.88 | -6.39 | 0.60 |
| cond@PhaseNC | -13.95 | -18.60 | -10.24 |
| cond@SexW | -1.14 | -4.34 | 1.16 |
| cond@Age.z | 0.11 | -1.30 | 1.80 |
| cond@Session.z | 0.14 | -0.57 | 0.90 |
| cond@SpeciesWolf:PhaseNC | -2.70 | -6.44 | 1.92 |

**Latency to get in apparatus proximity**

Surv_prox <- coxme(Surv(Pre_Latency.to.get.in.proximity__Total.duration, Pre_Latency.to.get.in.proximity__Binary) ~ Species * Phase + Sex + Session + Age.z + (1 + Session.z|AnimalID), data = C_all_data)

**Full-null model comparison:**

|  | Df | LogLik | Df | Chisq | Pr(>Chisq) |
| --- | --- | --- | --- | --- | --- |
| Full | 33.28 | -1542.00 |  |  |  |
| Null | 34.66 | -1540.00 | 1.37 | 3.973 | 0.05 |

**Model summary:**

|  | coef | exp(coef) | se(coef) | z | p |
| --- | --- | --- | --- | --- | --- |
| SpeciesWolf | -2.04 | 0.13 | 0.41 | -4.99 | 0.00 |
| PhaseNC | 0.09 | 1.09 | 0.18 | 0.49 | 0.62 |
| SexW | 0.50 | 1.64 | 0.37 | 1.36 | 0.17 |
| Session | 0.00 | 1.00 | 0.06 | 0.06 | 0.95 |
| Age.z | 0.26 | 1.30 | 0.20 | 1.32 | 0.19 |
| SpeciesWolf:PhaseNC | 0.10 | 1.10 | 0.25 | 0.39 | 0.70 |

**Confidence intervals:**

|  | Estimate | Lower | Upper |
| --- | --- | --- | --- |
| SpeciesWolf | -2.04 | -2.84 | -1.24 |
| PhaseNC | 0.09 | -0.27 | 0.45 |
| SexW | 0.50 | -0.22 | 1.21 |
| Session | 0.00 | -0.12 | 0.12 |
| Age.z | 0.27 | -0.13 | 0.66 |
| SpeciesWolf:PhaseNC | 0.10 | -0.39 | 0.58 |

**Latency to start pulling the rope**

Latency to pull the rope (Species-effect (Dogs vs wolves): β ± SE = -1.24 ± 0.54; *p*=0.02), regardless of previous conflict (Figure 2b).

surv_pull <- coxme(Surv(Pre_Latency.to.start.pulling__Total.duration, Pre_Latency.to.start.pulling__Binary) ~ Species * Phase + Sex + Session + Age.z + (1+Session.z|AnimalID), data = NCa_dat)

**Full-null model comparison:**

|  | Df | LogLik | Df | Chisq | Pr(>Chisq) |  |
| --- | --- | --- | --- | --- | --- | --- |
| Full | 29.47 | -1222.70 |  |  |  |  |
| Null | 29.79 | -1222.00 | 0.32 | 1.32 | <0.001 | *** |

**Model summary:**

|  | coef | exp(coef) | se(coef) | z | p |
| --- | --- | --- | --- | --- | --- |
| SpeciesWolf | -1.21 | 0.30 | 0.54 | -2.23 | 0.03 |
| PhaseNC | 0.25 | 1.28 | 0.22 | 1.10 | 0.27 |
| SexW | -0.01 | 0.99 | 0.50 | -0.03 | 0.98 |
| Session | 0.12 | 1.13 | 0.05 | 2.27 | 0.02 |
| Age.z | 0.09 | 1.10 | 0.27 | 0.35 | 0.73 |
| SpeciesWolf:PhaseNC | -0.19 | 0.83 | 0.32 | -0.61 | 0.54 |

**Confidence intervals:**

|  | Estimate | Lower | Upper |
| --- | --- | --- | --- |
| SpeciesWolf | -1.21 | -2.27 | -0.15 |
| PhaseNC | 0.25 | -0.19 | 0.69 |
| SexW | -0.01 | -0.99 | 0.96 |
| Session | 0.12 | 0.02 | 0.23 |
| Age.z | 0.094 | -0.44 | 0.63 |
| SpeciesWolf:PhaseNC | -0.19 | -0.81 | 0.43 |

**Duration Tail wagging**

C_model_Tail <- glmmTMB(Pre_Tail.wagging_PropT_Total.duration ~ Species * Phase + Sex + Session + Food+ Age.z + (1 + Food.H + Session.z||AnimalID) , data = Tail.data, family=beta_family)

**Full-null model comparison:**

|  | Df | AIC | BIC | logLik | deviance | Chisq Chi | Df | Pr(>Chisq) |  |
| --- | --- | --- | --- | --- | --- | --- | --- | --- | --- |
| C_model_Tail.0 | 9 | -1488.10 | -1453.50 | 753.07 | -1506.1 |  |  |  |  |
| C_model_Tail | 12 | -1501.30 | -1455.10 | 762.64 | -1525.3 | 19.15 | 3 | 2.54E-04 | *** |

**Model summary:**

|  | Estimate | Std. Error | z-value | Pr(>\|z\|) |  |
| --- | --- | --- | --- | --- | --- |
| (Intercept) | -0.52 | 0.47 | -1.12 | 0.26 |  |
| SpeciesWolf | -2.16 | 0.52 | -4.13 | 0.00 | *** |
| PhaseNC | -0.35 | 0.15 | -2.27 | 0.02 | * |
| SexW | -0.30 | 0.48 | -0.62 | 0.54 |  |
| Session | 0.04 | 0.06 | 0.74 | 0.46 |  |
| FoodH | -0.01 | 0.10 | -0.08 | 0.93 |  |
| Age.z | -0.31 | 0.26 | -1.17 | 0.24 |  |
| SpeciesWolf:PhaseNC | 0 | 0.22 | 0.72 | 0.47 |  |

**Confidence intervals:**

|  | orig | X2.5. | X97.5. |
| --- | --- | --- | --- |
| cond@(Intercept) | -0.52 | -1.45 | 0.39 |
| cond@SpeciesWolf | -2.16 | -3.29 | -1.12 |
| cond@PhaseNC | -0.35 | -0.64 | -0.03 |
| cond@SexW | -0.30 | -1.21 | 0.63 |
| cond@Session | 0.04 | -0.08 | 0.15 |
| cond@FoodH | -0.01 | -0.21 | 0.20 |
| cond@Age.z | -0.31 | -0.83 | 0.23 |
| cond@SpeciesWolf:PhaseNC | 0.16 | -0.29 | 0.60 |
| disp@(Intercept) | 1.44 | 1.28 | 1.65 |

**Post-hoc:**

| contrast | estimate | SE | df | z.ratio | p.value |
| --- | --- | --- | --- | --- | --- |
| D - W | 2.11 | 0.517 | Inf | 4.078 | <.0001 |
| C – NC | 0.27 | 0.11 | Inf | 2.42 | 0.016 |

**Looking at the human**

C_model_GazeH <- glmmTMB(Pre_Gazing.towards.conflict.partner_PropT_~ Species * Phase + Sex + Session + Food+ Age.z + (1 + Session.z||AnimalID) , data = GazeH.data, family=beta_family)

**Full-null model comparison:**

|  | Df | AIC | BIC | logLik | deviance | Chisq Chi | Df | Pr(>Chisq) |  |
| --- | --- | --- | --- | --- | --- | --- | --- | --- | --- |
| C_model_GazeH.0 | 8 | -1002.30 | -971.52 | 509.14 | -1018.3 |  |  |  |  |
| C_model_GazeH | 11 | -1008.70 | -966.39 | 515.35 | -1030.7 | 12.415 | 3 | <0.001 | ** |

**Model summary:**

|  | Estimate | Std. Error | z-value | Pr(>\|z\|) |  |
| --- | --- | --- | --- | --- | --- |
| (Intercept) | -2.09 | 0.18 | -11.41 | <0.001 | *** |
| SpeciesWolf | -0.65 | 0.17 | -3.81 | 0.00 | *** |
| PhaseNC | -0.18 | 0.14 | -1.22 | 0.22 |  |
| SexW | -0.05 | 0.14 | -0.34 | 0.73 |  |
| Session | -0.04 | 0.03 | -1.29 | 0.20 |  |
| FoodH | 0.67 | 0.10 | 6.84 | 0.00 | *** |
| Age.z | 0.02 | 0.08 | 0.23 | 0.82 |  |
| SpeciesWolf:PhaseNC | 0 | 0.21 | 0.83 | 0.40 |  |

**Confidence intervals:**

|  | orig | X2.5. | X97.5. |
| --- | --- | --- | --- |
| cond@(Intercept) | -2.09 | -2.45 | -1.73 |
| cond@SpeciesWolf | -0.65 | -0.99 | -0.30 |
| cond@PhaseNC | -0.18 | -0.44 | 0.11 |
| cond@SexW | -0.05 | -0.34 | 0.24 |
| cond@Session | -0.04 | -0.11 | 0.03 |
| cond@FoodH | 0.67 | 0.49 | 0.86 |
| cond@Age.z | 0.02 | -0.14 | 0.17 |
| cond@SpeciesWolf:PhaseNC | 0.17 | -0.21 | 0.56 |
| disp@(Intercept) | 2.09 | 1.95 | 2.29 |

**Post-hoc:**

| contrast | estimate | SE | df | z.ratio | p.value |
| --- | --- | --- | --- | --- | --- |
| Dog - Wolf | 0.59 | 0.156 | Inf | 3.783 | 0.0002 |
| A - H | -0.67 | 0.0987 | Inf | -6.825 | <.0001 |

## **STATISTICAL ANALYSIS – Animals – Post Phase**

**Self-directed behaviours**

PC_model_Displacement <- glmmTMB(Post.Displacement.Binary ~ Species * Phase + Sex + Food + Session + Age.z + (1 + Food.H + Session.z || AnimalID)+ offset(log(Post_Sum.proximity.to.human__Trial.duration)), data = P.Displacement.data, familly = binomial(link = "logit"))

**Full-null model comparison:**

|  | Df | AIC | BIC | logLik | deviance | Chisq Chi | Df | Pr(>Chisq) |  |
| --- | --- | --- | --- | --- | --- | --- | --- | --- | --- |
| PC_model_Displacement.0 | 8 | 659.74 | 690.62 | -321.87 | 643.74 |  |  |  |  |
| PC_model_Displacement | 11 | 628.92 | 671.39 | -303.46 | 606.92 | 36.813 | 3 | 5.04E-08 | *** |

**Reduced model summary:**

|  | Estimate | Std. Error | z-value | Pr(>\|z\|) |  |
| --- | --- | --- | --- | --- | --- |
| (Intercept) | -3.74 | 0.53 | -7.08 | 0.00 | *** |
| SpeciesWolf | 1.58 | 0.36 | 4.36 | 0.00 | *** |
| PhaseNC | 1.35 | 0.31 | 4.43 | 0.00 | *** |
| SexW | -0.29 | 0.33 | -0.87 | 0.38 |  |
| FoodH | -0.50 | 0.33 | -1.55 | 0.12 |  |
| Session | 0.11 | 0.13 | 0.86 | 0.39 |  |
| Age.z | 0.05 | 0.18 | 0.27 | 0.79 |  |

**Confidence intervals:**

|  | orig | X2.5. | X97.5. |
| --- | --- | --- | --- |
| cond@(Intercept) | -3.74 | -4.75 | -2.69 |
| cond@SpeciesWolf | 1.58 | 0.84 | 2.36 |
| cond@PhaseNC | 1.35 | 0.97 | 2.12 |
| cond@SexW | -0.29 | -0.77 | 0.37 |
| cond@FoodH | -0.50 | -1.07 | 0.17 |
| cond@Session | 0.11 | -0.09 | 0.33 |
| cond@Age.z | 0.05 | -0.34 | 0.35 |

**Post-hoc:**

| contrast | estimate | SE | df | z.ratio | p.value |
| --- | --- | --- | --- | --- | --- |
| Dog - Wolf | -1.58 | 0.36 | Inf | -4.36 | <.0001 |
| C - NC | -1.35 | 0.31 | Inf | -4.43 | <.0001 |

**Adverse behaviours**

PC_model_Avers.n.D <- glmmTMB(Post_AdvNonDispl ~ Species * Phase + Sex + Food + Session + Age.z + (1 + Food.H + Session.z || AnimalID)+ offset(Post_Sum.proximity.to.human__Trial.duration), data = P.Adv.nD.data, family = binomial(link = "logit"))

**Full-null model comparison:**

|  | Df | AIC | BIC | logLik | deviance | Chisq Chi | Df | Pr(>Chisq) |  |
| --- | --- | --- | --- | --- | --- | --- | --- | --- | --- |
| PC_model_Avers.n.D.0 | 8 | 178.28 | 209.17 | -81.14 | 162.28 |  |  |  |  |
| PC_model_Avers.n.D | 11 | 181.92 | 224.39 | -79.96 | 159.92 | 2.37 | 3 | 0.50 |  |

**Confidence intervals:**

|  | orig | X2.5. | X97.5. |
| --- | --- | --- | --- |
| cond@(Intercept) | -10.55 | -29.49 | -6.42 |
| cond@SpeciesWolf | 2.50 | -3.50 | 13.51 |
| cond@PhaseNC | 1.15 | -20.09 | 12.22 |
| cond@SexW | -0.06 | -4.62 | 4.13 |
| cond@FoodH | 0.35 | -1.41 | 3.51 |
| cond@Session | 0.14 | -0.68 | 0.84 |
| cond@Age.z | 1.24 | -1.41 | 3.02 |
| cond@SpeciesWolf:PhaseNC | -1.67 | -13.64 | 18.71 |

**Tail wagging**

PC_model_PC.Tail <- glmmTMB(Post_Tail.wagging_PropT ~ Species * Phase + Food + Sex + Session + Age.z + (1 + Food.H + Session.z||AnimalID) , data = PC.Tail.data, family=beta_family)

**Full-null model comparison:**

|  | Df | AIC | BIC | logLik | deviance | Chisq Chi | Df | Pr(>Chisq) |  |
| --- | --- | --- | --- | --- | --- | --- | --- | --- | --- |
| PC_model_PC.Tail.0 | 8 | -1846.00 | -1815.40 | 930.97 | -1862 |  |  |  |  |
| PC_model_PC.Tail | 12 | -1865.20 | -1819.40 | 944.60 | -1889.2 | 27.256 | 4 | <0.001 | *** |

**Model summary:**

|  | Estimate | Std. Errror | z-value | Pr(>\|z\|) |  |
| --- | --- | --- | --- | --- | --- |
| (Intercept) | -2.34 | 0.20 | -11.77 | <0.001 | *** |
| SpeciesWolf | -0.99 | 0.18 | -5.47 | 0.00 | *** |
| PhaseNC | 0.14 | 0.15 | 0.96 | 0.34 |  |
| FoodH | 0.12 | 0.10 | 1.13 | 0.26 |  |
| SexW | -0.21 | 0.15 | -1.47 | 0.14 |  |
| Session | -0.09 | 0.04 | -2.08 | 0.04 | * |
| Age.z | -0.02 | 0.08 | -0.26 | 0.80 |  |
| SpeciesWolf:PhaseNC | 0 | 0.22 | -0.39 | 0.70 |  |

**Confidence intervals:**

|  | orig | X2.5. | X97.5. |
| --- | --- | --- | --- |
| cond@(Intercept) | -2.34 | -2.74 | -1.96 |
| cond@SpeciesWolf | -0.99 | -1.33 | -0.64 |
| cond@PhaseNC | 0.14 | -0.15 | 0.45 |
| cond@FoodH | 0.12 | -0.09 | 0.31 |
| cond@SexW | -0.21 | -0.49 | 0.05 |
| cond@Session | -0.09 | -0.16 | -0.01 |
| cond@Age.z | -0.02 | -0.18 | 0.13 |
| cond@SpeciesWolf:PhaseNC | -0.09 | -0.57 | 0.32 |
| disp@(Intercept) | 2.25 | 2.08 | 2.48 |

**Post-hoc:**

| contrast | estimate | SE | df | z.ratio | p.value |
| --- | --- | --- | --- | --- | --- |
| Dog - Wolf | 1.02 | 0.163 | Inf | 6.215 | <.0001 |

**Latency to approach the human**

surv_proxH <- coxme(Surv Post_Latency.to.approach.conflict.partner.after.conflict_Corr_Total.duration, Post_Latency.to.approach.conflict.partner.after.conflict_Binary_) ~ Species * Trial + Food + Sex + Session + Age.z + (1+Session.z|AnimalID), data = C_all_data)

**Full-null model comparison:**

|  | Df | LogLik | Df | Chisq | Pr(>Chisq) |  |
| --- | --- | --- | --- | --- | --- | --- |
| full | 24.57 | -1488.80 |  |  |  |  |
| null | 22.59 | -1503.70 | -1.98 | 29.84 | 3.32E-07 | *** |

**Model summary:**

|  | Estimate | Std. Error | z-value | Pr(>\|z\|) |  |
| --- | --- | --- | --- | --- | --- |
| (Intercept) | -1.84 | 0.21 | -8.73 | <0.001 | *** |
| SpeciesWolf | -0.64 | 0.21 | -3.09 | 0.00 | ** |
| PhaseNC | -0.17 | 0.16 | -1.06 | 0.29 |  |
| FoodH | 0.23 | 0.11 | 2.18 | 0.02 | * |
| SexW | -0.18 | 0.18 | -1.03 | 0.30 |  |
| Session | -0.08 | 0.04 | -1.89 | 0.06 | . |
| Age.z | 0.11 | 0.10 | 1.08 | 0.28 |  |
| SpeciesWolf:PhaseNC | 0.15 | 0.23 | 0.68 | 0.49 |  |

**Confidence intervals:**

|  | Estimate | Lower | Upper |
| --- | --- | --- | --- |
| SpeciesWolf | -1.10 | -1.62 | -0.58 |
| TrialC2 | 0.25 | -0.14 | 0.64 |
| TrialNC | -0.18 | -0.57 | 0.21 |
| FoodH | 0.58 | 0.34 | 0.82 |
| SexW | -0.07 | -0.44 | 0.29 |
| Session | -0.08 | -0.18 | 0.02 |
| Age.z | 0.10 | -0.11 | 0.30 |
| SpeciesWolf:TrialC2 | 0.13 | -0.44 | 0.69 |
| SpeciesWolf:TrialNC | 0.38 | -0.19 | 0.95 |

**Post-hoc:**

| contrast | estimate | SE | df | z.ratio | p.value |
| --- | --- | --- | --- | --- | --- |
| Dog - Wolf | 0.93 | 0.203 | Inf | 4.594 | <.0001 |
| C1 - C2 | -0.31 | 0.14 | Inf | -2.146 | 0.08 |
| C1 - NC | 0.01 | 0.145 | Inf | 0.035 | 1.0 |
| C2 - NC | 0.31 | 0.14 | Inf | 2.175 | 0.08 |
| A - H | -0.59 | 0.121 | Inf | -4.847 | <.0001 |

**Duration in proximity (< 1 body length)**

PC_model_CProx <- glmmTMB(Post_Close.proximity.to.human__PropT ~ Species * Phase + Food + Sex + Session + Age.z + (1 + Food.H + Session.z||AnimalID) , data = CProx.data, family=beta_family)

**Full-null model comparison:**

|  | Df | AIC | BIC | logLik | deviance | Chisq Chi | Df | Pr(>Chisq) |  |
| --- | --- | --- | --- | --- | --- | --- | --- | --- | --- |
| PC_model_CProx.0 | 8 | -1196.8 | -1166.3 | 606.41 | -1212.8 |  |  |  |  |
| PC_model_CProx | 15 | -1197.30 | -1140.10 | 613.67 | -1227.3 | 14.516 | 1 | 0.04 | * |

**Model summary:**

|  | Estimate | Std. Error | z-value | Pr(>\|z\|) |  |
| --- | --- | --- | --- | --- | --- |
| (Intercept) | -1.81 | 0.23 | -8.02 | 1.06E-15 | *** |
| (Intercept) | -1.84 | 0.21 | -8.73 | <0.001 | *** |
| SpeciesWolf | -0.64 | 0.21 | -3.09 | 0.00 | ** |
| PhaseNC | -0.17 | 0.16 | -1.06 | 0.29083 |  |
| FoodH | 0.23 | 0.11 | 2.18 | 0.02927 | * |
| SexW | -0.18 | 0.18 | -1.03 | 3.05E-01 |  |
| Session | -0.08 | 0.04 | -1.89 | 0.06 | . |
| Age.z | 0.11 | 0.10 | 1.08 | 0.28 |  |

**Confidence intervals:**

|  | Orig | X2.5 | X97.5 |
| --- | --- | --- | --- |
| cond@(Intercept) | -1.8 | -2.23 | -1.37 |
| cond@SpeciesWolf | -0.70 | -1.18 | -0.20 |
| cond@FoodH | 0.17 | -0.17 | 0.52 |
| cond@PhaseNC | -0.19 | -0.64 | 0.28 |
| cond@SexW | -0.18 | -0.53 | 0.20 |
| cond@Session | -0.08 | -0.16 | 0.00 |
| cond@Age.z | 0.11 | -0.09 | 0.30 |
| cond@SpeciesWolf:FoodH | 0.11 | -0.42 | 0.62 |
| cond@SpeciesWolf:PhaseNC | 0.17 | -0.47 | 0.83 |
| cond@FoodH:PhaseNC | 0.04 | -0.61 | 0.72 |
| cond@SpeciesWolf:FoodH:PhaseNC | -0.03 | -0.94 | 0.85 |
| disp@(Intercept) | 1.70 | 1.56 | 1.95 |

**Post-hoc:**

| contrast | estimate | SE | df | z.ratio | p.value |
| --- | --- | --- | --- | --- | --- |
| Dog - Wolf | 0.59 | 0.19 | Inf | 3.06 | 0.02 |
| A - H | -0.23 | 0.11 | Inf | -2.19 | 0.03 |

**Gazing at the human**

PC_model_GazeH.bin <- glmmTMB(Post_GazeHuman_Bin ~ Species * Phase + Sex + Food + Session + Age.z + (1 + Food.H + Session.z || AnimalID), data = PC.GazeH, family = binomial(link = "logit"))

**Full-null model comparison:**

|  | Df | AIC | BIC | logLik | deviance | Chisq Chi | Df | Pr(>Chisq) |  |
| --- | --- | --- | --- | --- | --- | --- | --- | --- | --- |
| PC_model_GazeH.bin.0 | 8 | 171.19 | 201.73 | -77.596 | 155.19 |  |  |  |  |
| PC_model_GazeH.bin | 11 | 163.65 | 205.63 | -70.823 | 141.65 | 13.55 | 3 | <0.001 | ** |

**Model summary:**

|  | Df | AIC | LRT | Pr(>Chi) |  |
| --- | --- | --- | --- | --- | --- |
| <none> | 163.65 |  |  |  |  |
| Sex | 1.00 | 163.93 | 2.28 | 1.31E-01 |  |
| Food | 1.00 | 172.41 | 10.77 | 0.00 | ** |
| Session | 1.00 | 163.53 | 1.88 | 0.17 |  |
| Age.z | 1.00 | 178.74 | 17.09 | 0.00 | *** |
| Species:Phase | 1.00 | 162.06 | 0.41 | 0.52 |  |

**Confidence intervals:**

|  | orig | X2.5. | X97.5. |
| --- | --- | --- | --- |
| cond@(Intercept) | 5.07 | 3.59 | 9.02 |
| cond@SpeciesWolf | -2.05 | -4.33 | -0.83 |
| cond@PhaseNC | -0.38 | -2.33 | 1.93 |
| cond@SexW | -0.78 | -2.26 | 0.25 |
| cond@FoodH | 1.66 | 0.76 | 3.59 |
| cond@Session | -0.30 | -0.85 | 0.11 |
| cond@Age.z | 1.12 | 0.64 | 2.02 |
| cond@SpeciesWolf:PhaseNC | 0.66 | -2.34 | 3.52 |

**Post-hoc:**

| contrast | estimate | SE | df | z.ratio | p.value |
| --- | --- | --- | --- | --- | --- |
| Dog - Wolf | 1.81 | 0.53 | Inf | 3.4 | 0.00 |
| A - H | -1.65 | 0.54 | Inf | -3.074 | 0.0021 |
